# Exploring the Effect of an Obesity-Prevention Intervention on Various Child Subgroups: A Post Hoc Subgroup Analysis of the Kiel Obesity Prevention Study

**DOI:** 10.3390/nu16183220

**Published:** 2024-09-23

**Authors:** Elizabeth Mannion, Kristine Bihrmann, Sandra Plachta-Danielzik, Manfred J. Müller, Anja Bosy-Westphal, Christian Ritz

**Affiliations:** 1 National Institute of Public Health, University of Southern Denmark, 1455 Copenhagen, Denmark; elman@sdu.dk (E.M.); akri@sdu.dk (K.B.); 2 Competence Network for IBD, 24103 Kiel, Germany; s.plachta-danielzik@kompetenznetz-darmerkrankungen.de; 3 Institute of Human Nutrition and Food Science, Christian-Albrechts University of Kiel, 24118 Kiel, Germany; mmueller@nutrfoodsc.uni-kiel.de (M.J.M.); abosyw@nutrition.uni-kiel.de (A.B.-W.)

**Keywords:** childhood obesity, subgroup analysis, dietary intervention, physical activity, obesity prevention

## Abstract

Background: This study investigated potential subgroups of children within the Kiel Obesity Prevention Study (KOPS) for differing treatment effects for the outcome measures of overweight or obesity at 4 years. The KOPS study delivered a multicomponent school intervention to cohorts of children in Kiel but found no overall effect on the weight status outcome. However, KOPS authors suggested there may be subgroup variations in treatment effect. Data were collected as part of the KOPS for samples of 6-year-olds between 1996 and 2001, with 4-year follow-up measurements between 2000 and 2004. Methods: The present study conducted a post hoc subgroup analysis of the odds of obesity or overweight at 4-year follow-up compared to normal weight (*n* = 1646). A generalized linear mixed-effects model, including a treatment–subgroup interaction term, was used to estimate subgroups as a moderator of the treatment effects on the outcomes of obesity or overweight at 4-year follow-up. Results: The findings indicated several subgroup–treatment interaction effects relating to physical activity indicators. TV or PC not being one of a child’s top 3 activities at baseline was associated with a significantly decreased odds ratio of obesity at 4 years in the intervention group (OR, 0.04; 95% CI, 0.004 to 0.45) compared to the non-intervention group (OR, 0.96; 95% CI, 0.29 to 3.14), *p* = 0.02. Weekly activity in a sports club at baseline was associated with a decreased odds ratio of overweight at 4 years in the intervention group (OR, 0.38; 95% CI, 0.16 to 0.85) compared to the non-intervention group (OR, 0.91; 95% CI, 0.70 to 1.17). This was a significant difference (*p* = 0.04). Conclusions: These findings suggest that children’s baseline physical activity may impact treatment effects on the outcomes of overweight and obesity, creating opportunities to increase the effectiveness of interventions on preventing obesity.

## 1. Introduction

The global prevalence of obesity among children and adolescents aged 5–19 has risen sharply from 2% in 1990 to just over 8% in 2022 [1]. Excess weight in childhood has short-term and long-term consequences. In the short term, children living with obesity are more likely to experience a range of adverse psychological conditions and social consequences, including depression, bullying, and discrimination [1,2]. In the long term, it is estimated that obesity is carried on into adulthood in 55% of children diagnosed as obese [3]. The age of onset of obesity impacts long-term health, with children who become obese before adolescence having a higher risk for developing type 2 diabetes and associated health complications earlier in life compared to those who gain excess weight later [1]. Adult obesity is associated with premature death and disability in adulthood and is a risk factor in the development of other non-communicable diseases, including diabetes, hypertension, and cardiovascular disease [1]. Obesity places a heavy economic and social burden on individuals, families, and nations [4].

The global childhood obesity epidemic has motivated a range of interventions to attenuate the prevalence of obesity among children. Within this context, school-based interventions have been promoted as a cost-effective initiative for reaching the most children and utilizing pre-existing infrastructure [1]. Schools offer an attractive setting for obesity-prevention interventions as children spend a significant amount of time there, consume multiple meals each week there, and have the opportunity to engage in organized physical activity. School-based interventions often include one or more of the following components: physical activity, diet, and education, with the potential to target three groups: children, parents, and teachers. However, it is common for school-based obesity-prevention studies to not find an overall significant long-term reduction in obesity-related outcomes [5]. This may be due to the determinants of obesity being complex and multifaceted, meaning a “one size fits all” approach delivered to all school children simultaneously produces heterogeneous treatment effects. Interventions that produce different treatment effects for certain subgroups of children risk deepening pre-existing health inequalities and outcomes, not least because they may be less cost-effective or ineffective [6]. Precision medicine has been proffered as a potential solution to address this problem. Precision medicine involves taking account of individual variability in genes, environment, and lifestyle when planning the best course of action for the prevention and treatment of obesity among different groups of people [7].

The Kiel Obesity Prevention Study (KOPS) ran longitudinally and delivered a multicomponent, school-based intervention to first graders in schools in the German city of Kiel. The study aimed to investigate the determinants of childhood excess weight and prevent obesity. Despite improving knowledge and health competencies, the study found no overall effect of the intervention on the mean BMI. However, KOPS authors suggested there may be subgroup variations in the treatment effect.

Against this background, the current study reanalyzed data collected as part of the KOPS study, with the aim of identifying subgroups of children with differing treatment effects for the outcome measures of overweight or obese weight status at 4-year follow-up. When subgroups of children are identified, it is possible to better tailor interventions to tackle childhood obesity without widening health inequalities. A complex systems lens is used to interpret the results found in this study, acknowledging that many health behaviors are often shaped and restricted by many mutually interactive variables within complex systems. It was hypothesized that subgroups with differing treatment effects would be found.

## 2. Materials and Methods

### 2.1. Study Population

The current study is a secondary data analysis study, using a sample of children who participated in the KOPS. Children included in this study had available data at both baseline and at 4-year follow-up. Only children who were categorized as “Normal weight”, “Overweight”, or “Obese” at 4-year follow-up were included in this study, excluding those who were “Underweight”. KOPS researchers implemented the school-based intervention in Kiel between 1996 and 2001, randomly assigning between two and four schools each year to receive the intervention [8,9]. Randomization was undertaken each year to provide all schools in Kiel (*N* = 32) an equal chance to receive the intervention; since there were limited resources, not all schools could participate at the same time, and because the intervention design meant that it was not possible to be repeated in schools by teachers [9]. This meant that intervention schools subsequently became non-intervention schools the following year.

### 2.2. Intervention

The intervention comprised healthy messages about diet and physical activity (eat fruit and vegetables every day, reduce intake of high-fat foods, keep active at least 1 h/d, and decrease television consumption to <1 h/d) communicated to children, parents, and teachers. The 6-year-olds were all addressed by 6 nutrition units, delivered by a nutritionist, over two to three weeks in their first year of school [9]. After each unit, running and physical games were offered for 20 min. Teachers were also trained with a half-day structured nutrition education program. The study was approved by the local ethics committee, and parental consent was given.

### 2.3. Outcomes

Obesity and overweight at 4-year follow-up were the outcome measures in this study. The Actual German BMI percentiles, used in the original study, were used to define weight classification [10]. These percentiles employed the following categories: underweight (≤10th percentile), normal weight (>10th to <90th percentile), overweight (≥90th to <97th percentile), and obesity (≥97th percentile). The study sample was split in two so outcome measures could be treated as binary variables. Children who were normal weight or obese formed one group, and children who were normal weight or overweight at 4-year follow-up formed the other. Children categorized as normal weight were therefore included in both groups as the reference.

### 2.4. Measurements

Anthropometric data were recorded at baseline and at 4-year follow-up in the original study. Measurements were collected by KOPS researchers on the school premises, face-to-face with children. Body composition measurements were taken, including waist and arm circumference, skin folds, height, and weight. Family demographics and characteristics information were collected in the original study using self-report survey questions answered by a parent or guardian. Baseline data included 140 variables with information on pre-natal characteristics, socio-economic indicators, family health history, and estimations of physical activity.

### 2.5. Statistical Analysis

Descriptive statistics were presented as mean and standard deviation (SD) or as a count and percentage for all variables at baseline, split by intervention group. For non-normally distributed data, median and IQR were reported. To determine between group differences at baseline, the non-parametric Chi-squared test for categorical variables, an independent samples *t*-test for continuous variables (with equal variance assumption), and the Mann–Whitney U test (for non-normally distributed variables) were used as part of the tableone package in R [11].

The comparison of associations between baseline characteristics and the effect of the intervention on the outcome measures, overweight and obesity at 4-year follow-up, were analyzed. A total of three analyses for each outcome measure were conducted: adjusted and unadjusted available case analysis and an adjusted analysis using pooled multiply imputed data. Available case analysis is a method that uses data that are available for the specific variables being analyzed, and therefore, sample size varies across analyses as some variables contain more missing data than others.

A generalized linear mixed model (GLMM) was used with the outcome of either obesity or overweight at 4-year follow-up. A treatment interaction was included, with each baseline variable and the intervention as fixed effects. Following this, an adjusted analysis was conducted, adjusting the model for age and sex. Schools were also included in the model as a random effect, incorporating and accounting for the possible school-to-school variability that may arise. The results were reported as separate odds ratios, and their 95% confidence intervals for each baseline characteristic were used for outcome measures, overweight and obesity at 4-year follow-up, by intervention group. A ratio of these odds ratios (RORs) was also presented with a corresponding 95% confidence interval and *p*-value. The ROR quantifies the difference in subgroups and between intervention groups for the treatment effect by comparing their respective odds ratios. A ROR of ≤0.8 indicates a possible decreased odds of overweight or obesity for the intervention group compared to the non-intervention group. Variables with a ROR of ≤0.8 were chosen as key results to be presented, despite not all being significant, due to the relevance to the research aims. The Bonferroni correction was applied to control for the increased risk of Type I errors due to multiple comparisons.

Analysis was then run on pooled multiply imputed datasets to compare effect sizes with the available case analysis to assess the impact of missing data. Multivariate Imputation by Chained Equations (MICE) was used to impute missing data in the dataset, with ten imputed datasets generated [12]. The ten imputed datasets were looped through the adjusted GLMM model, and the results were pooled using the mice package (version 3.16.0) in R [13]. The missing at random assumption was employed to utilize the correlations between variables in the dataset to generate imputed values. Random forests were used to determine variable importance in predicting obesity and overweight, including an interaction term for the intervention. Variables were ranked by their combined importance scores and presented by the difference in these scores between the intervention and non-intervention groups. All statistical analysis was performed using R version 4.3.3 [14]. The glmer() function in the lme4 package (version 1.1-35.5) in R was used to fit the linear mixed models, and looping was incorporated to fit the models with each baseline variable [15]. The level of significance for all analyses in this study was set to *p* < 0.05.

## 3. Results

### 3.1. Characteristics of the Study Population

A total of 1646 children were included in this study after excluding those with missing weight status data or those categorized as underweight at a 4-year follow-up. Within this sample, 319 children received the intervention, and 1327 did not. A total of 14 of the 32 schools in Kiel were assigned to be intervention schools during the period from 1996 to 2001. At 4-year follow-up, 83.66% (*n* = 1377) of children were categorized as normal weight, 11.42% (*n* = 188) as overweight, and 4.92% (*n* = 81) as obese. There was no significant difference in the sex split between the intervention and the non-intervention group. The mean age overall at baseline was 6.25 ± 0.36 years; this was not significantly different between groups. Table 1 presents the baseline characteristics of the study population for variables considered in later analysis as key findings, split by intervention group.

### 3.2. Comparison of Associations

#### 3.2.1. Overweight Results

Table 2 presents the key findings for the treatment effect by subgroup, where the odds ratio of overweight at 4-year follow-up is the outcome measure. Children who were sports club members at baseline had a smaller odds ratio of overweight in the intervention group compared to the non-intervention group, though this was not significant (ROR, 0.40; 95% CI, 0.15 to 1.13; *p* = 0.08). Furthermore, weekly sports club activity resulted in a significantly reduced odds ratio of overweight for those in the intervention group, where a 0.5 h increase resulted in a reduced odds ratio of overweight (ROR, 0.41; 95% CI, 0.18 to 0.97; *p* = 0.04). Children in the intervention group whose parents reported painting or cycling as one of their top three most frequent activities had a non-significantly reduced odds ratio of overweight (ROR, 0.65; 95% CI, 0.14 to 3.01; *p* = 0.59 and ROR, 0.61; 95% CI, 0.14 to 2.57; *p* = 0.50, respectively). Children of parents who reported that the TV or PC was not one of their child’s top three most frequent activities had a lower, but not significantly different, odds ratio of overweight in the intervention group (ROR, 0.27; 95% CI, 0.03 to 2.48; *p* = 0.25, respectively). Likewise, children in the intervention group whose parents reported their TV or PC usage was one hour or less per day had a smaller, but not significantly different, odds ratio of overweight compared to children with the same usage in the non-intervention group (ROR, 0.74; 95% CI, 0.05 to 11.32; *p* = 0.83). After Bonferroni correction, no *p* values remained significant. A full table of results for the outcome overweight can be found in Table A1, Appendix A. Variables excluded from the adjusted analysis due to a lack of data for the outcomes of overweight and obesity are listed in Table A3, Appendix B.

There were no notable differences in effect size between the unadjusted analysis and adjusted analysis when adjusting for age and sex (an unadjusted analysis for the outcome of overweight can be found in Table A4, Appendix C). Similarly, the results from the analysis on the pooled imputed datasets showed no meaningful observed differences in effect size between the pooled GLMM run on the multiply imputed datasets and the adjusted GLMM on the original, available-case dataset. The extent of missing data for some variables caused increased variability and wider 95% confidence intervals in the GLMM output from the pooled imputed datasets. The imputation technique (MICE) was unable to run when variables with high collinearity or no original data were present. Therefore, variables were removed from the dataset until the model ran. A total of 133 variables were included in the analysis after removing those with high collinearity or no original data. Imputations resulted in a total of 1458 observations for the obesity outcome model and 1565 observations for the overweight model. A full table of the results from the analysis of the pooled imputed datasets and a list of excluded variables can be found in Table A6, Table A7 and Table A8, Appendix D. The results from the random forest analysis for variable importance in predicting overweight and obesity, including an intervention interaction term, are presented in Supplementary Tables S1 and S2. The results presented are ranked by the difference in importance between intervention groups. The results indicate that baseline anthropometric measures were significant predictors of overweight and obesity at the 4-year follow-up.

**Figure 1:**
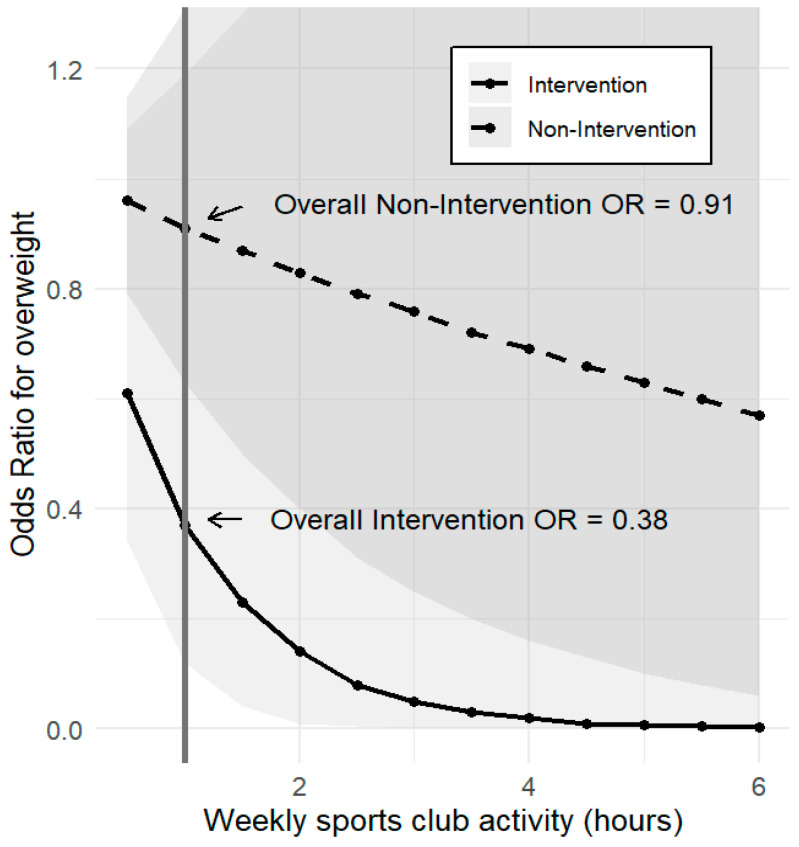
Odds ratio of overweight at 4-year follow-up for the intervention and non-intervention groups.

Figure 1 illustrates a dose–response relationship. The intervention group had a more rapid reduction in the odds ratio of overweight, for the same hours of weekly sports club activity, than the non-intervention group. The shaded areas show the 95% confidence intervals for each intervention group. The darkest shaded area indicates overlapping of the confidence intervals for the two groups.

#### 3.2.2. Obesity Results

The key findings for the treatment effect by subgroup are presented in Table 3, where the odds ratio of obesity at 4-year follow-up is the outcome measure. The odds ratio for obesity was reduced, but not significantly different, for children who were a sports club member at baseline and in the intervention group compared to those in the non-intervention group (ROR, 0.59; 95% CI, 0.10 to 3.60; *p* = 0.56). Cycling and painting, as one of the children’s top three activities, were also associated with a non-significantly decreased odds ratio of obesity for the intervention group (ROR, 0.43; 95% CI, 0.03 to 5.31; *p* = 0.51 and ROR, 0.42; 95% CI, 0.04 to 4.15; *p* = 0.46). Similarly, romping and swimming as a top three activity resulted in a decreased odds ratio of obesity for the intervention group, though not significant (ROR, 0.53; 95% CI, 0.04 to 6.55; *p* = 0.62 and ROR, 0.75; 95% CI, 0.06 to 10.29; *p* = 0.83). Children whose parents reported that the TV or PC was not one of their child’s top three activities had significantly different treatment effects. Those in the intervention group had a significantly reduced odds ratio of obesity compared to the non-intervention group (ROR, 0.04; 95% CI, 0.002 to 0.53; *p* = 0.02). Similar to the overweight analysis, no *p*-values remained significant after applying the Bonferroni correction. A full table of results for the outcome of obesity can be found in Table A2, Appendix A. As with the overweight model, the results were unaltered when adjusted for age and sex (unadjusted results for the outcome of obesity can be found in Table A5, Appendix C).

## 4. Discussion

The current study builds on the original KOPS study by investigating the treatment effects of the school-based intervention in subgroups of the study sample. While the original study found no overall significant change in weight status after 4 years, the results from the current study showed differing treatment effects on the odds of overweight and obesity between the two intervention groups within several subgroups based on indicators of baseline physical activity. Both studies identified a social gradient in the treatment effect when overweight was the outcome; children from middle and high socio-economic status (SES) families had lower odds of being overweight, with the lowest odds in the high SES group. In contrast to the original study, which found no significant effect on obesity, this study found that less sedentary behavior was significantly linked to a reduced odds ratio of obesity in the intervention group. These results suggest that school-based health promotion interventions can be effective in preventing obesity, especially when considering SES and physical activity levels.

### 4.1. Physical Activity and Sedentary Behavior—A Dose–Response Relationship

It is known that physical activity has the potential effect of preventing childhood obesity by increasing energy expenditure. In Europe, less than 50% of children meet the WHO’s recommended 60 min of moderate-to-vigorous (MVPA) physical activity per day [16]. The current literature emphasizes the importance of the intensity of physical activity on health outcomes, with numerous authors reporting that MVPA is associated with improved health outcomes, while the effect of light physical activity (LPA) has even been negatively associated with the risk of overweight and obesity in children who meet the recommended physical activity time guidelines [17,18].

The results from the current study may suggest that the intervention group was exposed to a higher dose of MVPA via the physical activity aspects of the intervention. This dose–response relationship may explain the observed reduced odds ratios of overweight and obesity for the intervention group for subgroups such as weekly activity in a sports club. One explanation could be that the intervention group’s exposure to the intervention prompted a higher dose of MVPA outside of sports clubs, pushing the intervention group over an undefined threshold to illicit significant weight-management benefits at fewer hours of weekly sports club activity than the non-intervention group.

Physical activity levels in children have been found to be associated with sedentary behaviors such as television, computer, and social media use. Grier et al. found an association between television viewing of more than 2 h a day and decreased fitness [19]. Time spent engaging in sedentary behavior in children not only displaces the amount of time able to be spent performing physical activity but has been found to be associated with worse sleep patterns, unhealthy snacking behavior, and increased exposure to unhealthy food marketing—all of which are associated with an increased risk of overweight and obesity [20,21,22]. Previous research has found a dose–response relationship between screen time and the risk of overweight or obesity, where those with the highest screen time have the highest risk of overweight or obesity [23,24]. Media use has also been identified as a significant mediator in the relationship between socio-economic status and fat mass in 5–7-year-olds [25]. The results from this current study found that TV or PC usage reported as not being one of a child’s top three activities was associated with a significantly reduced odds ratio of obesity in the intervention group compared to the non-intervention group. However, it should be noted that the absence of sedentary behavior does not necessarily mean the presence of increased physical activity. The results from the current study may be interpreted as the intervention group was exposed to the promotion of physical activity and other health-promotion behaviors more than the non-intervention group and may have spent their non-sedentary time more actively than the non-intervention group, resulting in the observed differing subgroup treatment effects. This notion of increased exposure to the promotion of physical activity for the intervention group may similarly explain the possible differing treatment effects in children who had cycling, swimming, romping, or painting as one of their top three activities by intervention group in the sense that these activities may have supported greater engagement in addition to increased exposure to physical activity promotion and less sedentary behavior as part of the intervention. However, these subgroups were not found to significantly differ between intervention groups in this study.

#### Parental Effects

There is some indication from the results of this study that parents may have an impact on their children’s odds of overweight and obesity. Though not statistically significantly different, the results from this study suggest there was a reduced odds ratio of overweight and obesity in the intervention group for the baseline variable of mothers’ weekly sports, while fathers’ weekly sports were associated with a reduced odds ratio of obesity in the intervention group, though also not significant. However, research suggests that the relationship between parents’ physical activity as a modeling behavior and levels of children’s physical activity is weak and somewhat inconsistent [26]. Other suggestions for the role that parents play in their child’s health behaviors include the theory of “self-efficacy”, which is a term coined by the behavioral psychologist Bandura [27]. Indeed, numerous studies have found that children whose parents report higher levels of self-efficacy are less likely to not meet physical activity recommendations and have higher levels of MVPA [28,29,30]. Furthermore, an association between parental self-efficacy and children’s screentime has been documented, with children of parents with lower self-efficacy exceeding the recommendation of no more than 2 h of screentime per day, significantly more than children whose parents had higher self-efficacy [31].

It is plausible that the KOPS intervention, through educating parents, increased their self-efficacy, which in turn may have improved their ability to support and promote healthier behaviors in their children. This may have contributed to some of the observed reduced odds ratios of overweight and obesity in the intervention group, even if the direct link to parental physical activity was not significant.

### 4.2. A Complex Systems Approach to Preventing Obesity

The current study aimed to identify subgroups of children with differing treatment effects of the KOPS intervention, with the view to better tailor interventions to tackle childhood obesity without widening health inequalities. The interpretation of these results has so far, and in many comparable studies, been discussed from an individual perspective, where levels of physical activity and sedentary behavior are implied to be a product of children’s or parental choice. However, several authors have called for obesity to be recognized as a part of a dynamic, complex system where individual behavior is shaped, enabled, and delimited by the wider social environment and non-linear causal mechanisms that may change over time [32,33].

In this context, to prevent the growing obesity epidemic, a more nuanced policy approach is warranted that addresses physical activity environments and barriers within a complex system. Such an approach could facilitate the practical application of the findings from this study, especially the significance of baseline physical activity levels, by creating environments supportive of their implementation. For instance, built and social environments have been found to influence children’s physical activity levels and should be modified at a national and community level. Living in walkable neighborhoods and having access to green space and leisure facilities are associated with higher levels of physical activity and lower levels of media use in children [34,35,36,37]. Children living in apartments, public housing, neighborhoods with high traffic levels or major roads, and areas lacking outdoor activity facilities have been found to have lower levels of physical activity and active transport [38,39,40].

### 4.3. Study Strengths and Limitations

One strength of the current study is its large sample size, which supports the ability to detect significantly different treatment effects across various subgroups. It is also a strength that the current study explored only priori given subgroups. This approach reduces the risk of p-hacking and, in turn, the likelihood of identifying patterns purely by chance, as the subgroups analyzed were based on criteria established during the design of the original KOPS.

The current study acknowledges the possibility of selection bias due to varying amounts of missing data by variable. If participants with incomplete data differed in baseline characteristics or weight outcomes from those with complete data, the available case analysis may not be representative of the population. Research shows that individuals with lower socio-economic status and worse health have higher non-response rates in health surveys [41]. To assess the impact of missing data, the researchers conducted an analysis on pooled imputed datasets, though the MICE method of imputation assumed that data were missing at random. The generalizability of these findings may be limited to settings similar to Kiel, Germany, such as school children in other urban areas in Western or European countries. In Southeast Asia, determinants of childhood obesity differ from those in developed Western countries, including factors such as access to clean water and varying cultural ideals about body image [42,43]. Given these geographical and cultural differences, other settings might exhibit different subgroups with distinct treatment effects.

Additionally, the duration of the intervention delivered as part of the KOPS may not have been sufficient to elicit the desired effects, as its components were delivered over 2 to 3 weeks. Recent research indicates that a longer, continuous school-based intervention of three years is optimal for achieving long-term obesity-prevention outcomes [44]. Furthermore, developments in obesity prevention highlight the usefulness of co-designing interventions with stakeholders, including children, to improve not only outcomes but also process measures such as motivation and retention [45]. Since the data analyzed in the current study were collected between 1996 and 2001, they do not reflect the implementation of this recent knowledge in intervention design. If motivational aspects and the duration of the KOPS were not at an optimal level, then it is expected that the intervention would not work for all children the same way. Consequently, it may be argued that the subgroup treatment effects observed in the current study reflect the shortcomings in the design of the KOPS. If the intervention were to be reproduced with the discussed updated design elements, it is possible that the observed subgroup treatment effects may differ.

Future research, in the form of randomized controlled trials, may usefully investigate subgroup-specific thresholds of physical activity and sedentary behavior needed to illicit the desired treatment of prevention effects in different environments and populations. Such trials may utilize objective measurements of physical activity, such as wearable activity trackers, to improve the accuracy of physical activity data.

## 5. Conclusions

The findings from the current study may be used as an evidence base to inform the design of future public health interventions since physical activity outside of and in combination with the intervention has been identified as an important factor in the treatment effect of interventions preventing overweight and obesity. Physical activity interventions may, therefore, be targeted at those children with limited outside intervention physical activity or with frequent sedentary habits to ensure they reach the threshold of time spent engaging in physical activity and reduce sedentary behavior as part of obesity-prevention programs.

## Figures and Tables

**Table 1:** Baseline characteristics of the 1646 children included in the study ^1^.

Characteristic	Intervention (*N* = 319) ^2^	Non-Intervention (*N* = 1327)	*p* ^3^
Anthropometric characteristics			
Age (years)	6.23 ± 0.36	6.25 ± 0.36	0.31
Male (%)	157 (49.2)	640 (48.2)	
Female (%)	162 (50.8)	687 (51.8)	0.80
Height (cm)	119.94 ± 5.31	120.31 ± 5.38	0.26
Weight (kg)	22.50 (21.00–24.55)	22.30 (20.70–25.00)	0.38
BMI (kg/m^2^)	15.71 (14.93–16.74)	15.53 (14.75–16.52)	0.03
BMI SDS	0.20 ± 0.91	0.11 ± 0.87	0.10
Fat mass percentage	21.28 (16.70–24.85)	20.83 (16.47–25.83)	0.38
Tricep skin fold (mm)	11.00 (9.15–14.00)	10.66 (9.00–13.30)	0.26
Sum of 4 skin folds (mm)	29.34 (24.74–38.28)	28.64 (23.00–36.34)	0.04
Waist circumference (cm)	55.00 (52.50–58.00)	55.00 (52.25–58.00)	0.37
Arm circumference (cm)	18.00 (17.50–19.50)	18.00 (17.00–19.50)	0.47
Physical activity characteristics			
Child is a sports club member (%)	154 (71.7)	492 (62.9)	0.02
Weekly sports club activity (hours)	1.50 (1.00–2.00)	1.00 (0.00–2.00)	0.01
Frequent activity: cycling (%)	45 (32.9)	223 (44.1)	0.02
Frequent activity: romping (%)	51 (37.2)	209 (41.1)	0.47
Frequent activity: painting (%)	93 (67.9)	348 (68.5)	0.97
Frequent activity: TV or PC (%)	18 (13.2)	104 (20.6)	0.07
TV/PC hours per day (hours)	1.00 (0.50–1.50)	1.00 (0.50–1.50)	0.19
Mother’s weekly sports (hours)	1.00 (0.00–2.00)	0.00 (0.00–2.00)	0.09
Father’s weekly sports (hours)	1.00 (0.00–3.00)	0.00 (0.00–2.00)	0.06
Parent’s activity is higher on weekends (%)	8 (25.8)	34 (29.83)	0.83
Socio-economic characteristics			
SES (%)			0.35
Low	36 (17.0)	154 (19.9)	
Middle	61 (28.8)	243 (31.4)	
High	115 (54.2)	377 (48.7)	
Mother graduated from school (%)			0.58
Grade 9	51 (24.1)	208 (27.1)	
Grade 10	75 (35.4)	274 (35.7)	
Grade 12	86 (40.5)	285 (37.2)	
Father graduated from school (%)			0.38
Grade 9	54 (28.7)	239 (33.5)	
Grade 10	44 (23.4)	163 (23.1)	
Grade 12	90 (47.9)	305 (43.4)	
Father has professional training (%)	118 (93.7)	440 (92.4)	0.78
Mother has professional training (%)	130 (95.6)	432 (85.5)	0.01
Mother is employed (%)	97 (45.4)	394 (50.23)	0.24
Father is employed (%)	183 (92.0)	673 (91.8)	>0.99
Single parent (%)	35 (16.1)	126 (16.1)	>0.99
Family’s health characteristics			
Mother is overweight (%)	35 (17.6)	163 (21.4)	0.17
Mother has elevated blood pressure (%)	21 (10.4)	64 (8.8)	0.57
Father had a heart attack (%)	6 (3.2)	22 (3.1)	>0.99
Portions of fruit per day	2.00 (1.00–2.00)	1.00 (1.00–2.00)	0.39
Number of meals eaten together	2.00 (1.00–2.00)	2.00 (1.00–2.00)	0.34
School should do more for health (%)	160 (86.5)	632 (89.7)	0.28

BMI SDS, Body Mass Index Standardized Deviation Score. SES, Socio-Economic Status. ^1^ Data are presented as mean ± standard deviation, count (percentage), or median (interquartile range) for non-normally distributed variables. ^2^ Counts (*n*) vary by variable due to missing data, so results are a mean, proportion, or median of the non-missing values. ^3^ *p*-value for test of group difference using an independent samples *t*-test for continuous data or Chi-square test for categorical data and Mann–Whitney U for non-normally distributed data.

**Table 2:** Treatment effect by subgroup, where the odds ratio of overweight at 4-year follow-up is the outcome measure.

Baseline Characteristic	Treatment ^1^						Treatment Baseline Interaction ^2^		
	Intervention			Non-Intervention					
Overweight	*N*	OR	95% CI	*N*	OR	95% CI	ROR	95% CI	*p*
Physical activity characteristics									
Child is a sports club member	209	0.27	(0.11, 0.67)	758	0.68	(0.42, 1.09)	0.40	(0.15, 1.13)	0.08
Weekly sports club activity (hours)	133	0.38	(0.16, 0.85)	484	0.91	(0.70, 1.17)	0.41	(0.18, 0.97)	0.04
Cycling is a top 3 activity	133	0.86	(0.21, 3.49)	487	1.32	(0.73, 2.40)	0.65	(0.14, 3.01)	0.59
Painting is a top 3 activity	133	0.43	(0.12, 1.58)	489	0.67	(0.36, 1.24)	0.61	(0.14, 2.57)	0.50
TV or PC is a top 3 activity (no)	132	0.86	(0.10, 7.28)	489	3.22	(1.73, 6.01)	0.27	(0.03, 2.48)	0.25
TV/PC one hour or less per day	76	0.45	(0.04, 5.16)	269	0.58	(0.18, 1.87)	0.74	(0.05, 11.32)	0.83
Mother’s weekly sports (hours)	131	0.59	(0.30, 1.15)	473	0.84	(0.66, 1.07)	0.69	(0.34, 1.41)	0.31
Socio-economic characteristics									
SES middle: low	206	0.45	(0.16, 1.31)	748	1.09	(0.58, 2.05)	0.42	(0.12, 1.44)	0.17
SES high: low	206	0.11	(0.03, 0.38)	748	0.55	(0.29, 1.04)	0.20	(0.05, 0.81)	0.03
Mother graduated from school, Grade 10:Grade 9	206	0.16	(0.05, 0.52)	741	0.84	(0.47, 1.48)	0.19	(0.05, 0.72)	0.01
Mother graduated from school, Grade 12:Grade 9	206	0.13	(0.04, 0.43)	741	0.44	(0.23, 0.84)	0.30	(0.08, 1.18)	0.09
Father graduated from school, Grade 12:Grade 9	183	0.10	(0.02, 0.48)	685	0.45	(0.24, 0.84)	0.23	(0.04, 1.26)	0.09
Mother has professional training	132	0.22	(0.02, 2.42)	486	0.40	(0.19, 0.82)	0.58	(0.05, 7.01)	0.67
Father is employed	192	0.29	(0.08, 1.02)	712	0.68	(0.31, 1.49)	0.41	(0.10, 1.82)	0.24
Single parent	208	0.50	(0.11, 2.25)	757	1.00	(0.512, 1.91)	0.52	(0.10, 2.66)	0.43
Family health characteristics									
Mother has elevated blood pressure	196	0.44	(0.06, 3.44)	706	2.00	(0.96, 4.14)	0.21	(0.02, 1.89)	0.16
Father had a heart attack	183	1.77	(0.20, 15.97)	687	2.14	(0.70, 6.55)	0.79	(0.06, 9.34)	0.85
Father’s alcohol use (three timesper week)	193	0.21	(0.03, 1.70)	709	0.46	(0.20, 1.08)	0.51	(0.05, 4.80)	0.60
Number of meals eaten together	211	0.87	(0.50, 1.50)	759	1.12	(0.82, 1.54)	0.79	(0.41, 1.49)	0.46
School should do more for health	178	0.58	(0.18, 1.91)	679	0.75	(0.36, 1.59)	0.74	(0.18, 3.02)	0.67

ROR, Ratio of Odds Ratios. ^1^ Data are presented as odds ratios and corresponding 95% confidence intervals for the outcome of obesity by treatment group. ^2^ A comparison of odds ratios between the treatment groups is presented as an ROR with corresponding 95% confidence intervals and *p*-values from a generalized linear mixed effects model (GLMM) adjusted for age and sex and including a treatment interaction term. School was included as the random effect.

**Table 3:** Treatment effect by subgroup, where the odds ratio of obesity at 4-year follow-up is the outcome measure.

Baseline Characteristic	Treatment ^1^						Treatment Baseline Interaction ^2^		
	Intervention			Non-Intervention					
Obesity	*N*	OR	95% CI	*N*	OR	95% CI	ROR	95% CI	*p*
Physical activity characteristics									
Child is a sports club member	193	0.33	(0.06 1.67)	709	0.56	(0.26, 1.23)	0.59	(0.10, 3.60)	0.56
Cycling is a top 3 activity	127	0.68	(0.067, 6.95)	458	1.54	(0.59, 4.04)	0.43	(0.03, 5.31)	0.51
Romping is a top 3 activity	127	0.61	(0.06, 6.32)	460	1.15	(0.43, 3.02)	0.53	(0.04, 6.55)	0.62
Painting is a top 3 activity	127	0.43	(0.06, 3.29)	460	0.94	(0.34, 2.6)	0.42	(0.04, 4.15)	0.46
Swimming is a top 3 activity	127	1.36	(0.12, 14.59)	459	2.04	(0.68, 6.10)	0.75	(0.06, 10.29)	0.83
TV or PC is a top 3 activity (no)	126	0.04	(0.004, 0.45)	460	0.96	(0.29, 3.14)	0.04	(0.002, 0.53)	0.02
Mother’s weekly sports (hours)	125	0.80	(0.39, 1.64)	450	1.02	(0.77, 1.34)	0.80	(0.37, 1.70)	0.56
Father’s weekly sports (hours)	113	0.83	(0.48, 1.44)	406	1.04	(0.82, 1.33)	0.77	(0.41, 1.45)	0.42
Parent’s activity is higher on weekends	29	2.86	(0.16, 52.05)	103	5.14	(0.45, 59.00)	0.54	(0.01, 24.18)	0.75
Socio-economic characteristics									
Mother has professional training	126	0.02	(<0.01, 0.28)	458	0.18	(0.07, 0.50)	0.12	(0.01, 1.60)	0.12
Father has professional training	117	0.16	(0.01, 2.50)	430	0.16	(0.04, 0.65)	0.72	(0.03, 15.20)	0.83
Mother is employed	192	0.58	(0.10, 3.25)	710	0.98	(0.44, 2.15)	0.58	(0.09, 3.85)	0.57
Family health characteristics									
Mother has elevated blood pressure	182	1.61	(0.17, 15.31)	660	3.28	(1.12, 9.62)	0.51	(0.04, 6.17)	0.59
Mother is overweight	179	1.04	(0.10, 10.47)	663	2.63	(1.07, 6.44)	0.38	(0.03, 4.58)	0.45
Portions of fruit per day	57	0.35	(0.02, 5.85)	133	2.27	(0.90, 5.72)	0.21	(0.01, 3.41)	0.27
Number of meals eaten together	196	0.37	(0.13, 1.05)	710	0.92	(0.55, 1.56)	0.38	(0.12, 1.22)	0.10
Anthropometric characteristics									
Arm circumference (cm)	290	2.20	(1.62, 3.00)	1167	3.65	(2.86, 4.65)	0.61	(0.42, 0.89)	0.01
Tricep skin fold (mm)	290	1.349	(1.18, 1.54)	1168	1.75	(1.57, 1.95)	0.76	(0.65, 0.90)	<0.01
Child’s BMI SDS	290	19.76	(19.56, 19.94)	1168	29.63	(29.35, 29.92)	0.65	(0.64, 0.66)	<0.01

ROR, Ratio of Odds Ratios. ^1^ Data are presented as odds ratios and corresponding 95% confidence intervals. for the outcome obesity by treatment group. ^2^ A comparison of odds ratios between the treatment groups is presented as an ROR with corresponding 95% confidence intervals and *p*-values from a generalized linear mixed effects model (GLMM) adjusted for age and sex and including a treatment interaction term. School was inputted as the random effect.

## Data Availability

The datasets presented in this article are not readily available because of ethical and privacy restrictions. Requests to access the datasets should be directed to the corresponding author.

## Supplementary Materials

The following supporting information can be downloaded at: https://www.mdpi.com/article/10.3390/nu16183220/s1, Table S1: Difference in combined variable importance scores between intervention groups when predicting overweight; Table S2: Difference in combined variable importance scores between intervention groups when predicting obesity.

## Appendix A

**Table A1:** Adjusted available case analysis—full results, overweight.

Baseline Characteristic	Treatment						Treatment Baseline Interaction		
	Intervention			Non-Intervention					
Overweight	*N*	OR	95% CI	*N*	OR	95% CI	ROR	95% CI	*p*
Anthropometric characteristics									
Weight (kg)	302	1.41	(1.25, 1.60)	1263	1.37	(1.30, 1.45)	1.04	(0.91, 1.19)	0.59
Height (cm)	302	1.09	(1.01, 1.17)	1263	1.05	(1.02, 1.09)	1.04	(0.96, 1.12)	0.36
Tricep skin fold (mm)	302	1.26	(1.13, 1.399)	1263	1.34	(1.27, 1.41)	0.94	(0.83, 1.052)	0.27
Bicep skin fold (mm)	302	1.31	(1.15, 1.50)	1263	1.33	(1.25, 1.41)	0.99	(0.85, 1.142)	0.86
Abdominal (mm)	302	1.26	(1.15, 1.389)	1263	1.33	(1.27, 1.39)	0.94	(0.85, 1.04)	0.23
SIF (mm)	302	1.19	(1.09, 1.309)	1263	1.30	(1.24, 1.36)	0.91	(0.83, 1.007)	0.07
SSF (mm)	302	1.45	(1.24, 1.69)	1263	1.41	(1.33, 1.51)	1.02	(0.86, 1.21)	0.81
Child sum of 4 skin folds	302	1.16	(1.05, 1.12)	1263	1.10	(1.09, 1.12)	0.98	(0.95, 1.02)	0.36
Arm circumference (cm)	302	2.03	(1.56, 2.65)	1262	2.14	(1.89, 2.42)	0.95	(0.71, 1.27)	0.72
Wasit (cm)	301	1.27	(1.16, 1.39)	1263	1.28	(1.23, 1.34)	0.99	(0.90, 1.10)	0.90
Hip circumference (cm)	301	1.17	(1.08, 1.27)	1263	1.24	(1.19, 1.29)	0.94	(0.86, 1.03)	0.19
Fat-free mass KOPS formula	297	1.47	(1.18, 1.77)	1233	1.36	(1.25, 1.47)	1.09	(0.88, 1.36)	0.44
Fat mass KOPS formula	297	1.78	(1.41, 2.05)	1233	1.67	(1.52, 1.83)	1.02	(0.83, 1.26)	0.85
Child’s fat-free mass(%)	297	0.85	(0.79, 0.91)	1233	0.88	(0.86, 0.91)	0.96	(0.89, 1.04)	0.32
Child’s fat mass (%)	297	1.22	(1.10, 1.26)	1233	1.13	(1.10, 1.17)	1.04	(0.96, 1.12)	0.32
Child’s BMI	302	2.51	(1.86, 3.47)	1263	2.55	(2.21, 2.95)	1.00	(0.71, 1.40)	0.98
Birth height (cm)	295	1.02	(0.89, 1.17)	1223	0.95	(0.90, 1.00)	1.08	(0.93, 1.25)	0.32
Child’s head circumference at birth	265	1.05	(0.81, 1.36)	1128	1.00	(0.90, 1.12)	1.05	(0.79, 1.39)	0.76
Height at 1 year (cm)	275	1.00	(0.87, 1.14)	1085	0.95	(0.89, 1.01)	1.05	(0.91, 1.22)	0.50
Height at 2 years (cm)	261	1.00	(0.90, 1.12)	1064	0.98	(0.93, 1.03)	1.02	(0.91, 1.15)	0.72
Physical activity characteristics									
Child is a sports club member	209	0.27	(0.11, 0.67)	758	0.68	(0.42, 1.09)	0.40	(0.15, 1.13)	0.08
Weekly sports club activity (hours)	133	0.38	(0.17, 0.85)	484	0.91	(0.70, 1.17)	0.41	(0.18, 0.97)	0.04
TV or PC per day (hours)	132	1.8	(0.75, 4.23)	480	2.00	(1.46, 2.75)	0.89	(0.36, 2.25)	0.81
Cycling is a top 3 activity	133	0.86	(0.21, 3.49)	487	1.32	(0.73, 2.40)	0.65	(0.14, 3.01)	0.59
Romping is a top 3 activity	133	1.79	(0.48, 6.32)	489	1.03	(0.56, 1.88)	1.77	(0.42, 7.44)	0.44
Painting is a top 3 activity	133	0.43	(0.12, 1.58)	489	0.67	(0.37, 1.24)	0.61	(0.14, 2.57)	0.50
Playing with toys is a top 3 activity	133	1.80	(0.48, 6.68)	489	0.95	(0.52, 1.74)	1.85	(0.43, 7.88)	0.41
Outdoor games are a top 3 activity	133	1.56	(0.43, 5.68)	489	1.11	(0.61, 2.01)	1.40	(0.33, 5.83)	0.65
Indoor games are a top 3 activity	133	0.92	(0.23, 3.76)	488	1.09	(0.57, 2.09)	0.85	(0.18, 4.01)	0.83
Swimming is a top 3 activity	133	2.00	(0.47, 8.21)	488	1.06	(0.48, 2.36)	1.81	(0.35, 9.34)	0.48
TV or PC is a top 3 activity (no)	132	0.86	(0.10, 7.28)	489	3.22	(1.73, 6.01)	0.27	(0.03, 2.48)	0.25
Mother’s weekly sports (hours)	131	0.59	(0.30, 1.15)	473	0.84	(0.66, 1.08)	0.69	(0.34, 1.41)	0.31
Father’s weekly sports (hours)	118	0.89	(0.62, 1.26)	435	1.04	(0.92, 1.19)	0.86	(0.59, 1.25)	0.42
Socio-economic characteristics									
Single parent	208	0.50	(0.11, 2.25)	757	1.00	(0.52, 1.91)	0.52	(0.10, 2.66)	0.43
Mother’s professional training	132	0.22	(0.02, 2.42)	486	0.4	(0.19, 0.82)	0.58	(0.05, 7.01)	0.67
Father’s professional training	122	0.41	(0.04, 4.02)	461	0.41	(0.16, 1.11)	1.06	(0.09, 12.62)	0.96
Mother is employed	208	0.81	(0.33, 1.98)	759	0.81	(0.50, 1.30)	0.99	(0.36, 2.75)	0.99
Father is employed	192	0.29	(0.08, 1.02)	712	0.68	(0.31, 1.49)	0.41	(0.09, 1.82)	0.24
Family’s health characteristics									
Mother’s age in years	298	0.95	(0.87, 1.04)	1250	0.98	(0.94, 1.01)	0.97	(0.89, 1.07)	0.57
Mother’s weight (kg)	297	1.02	(0.99, 1.05)	1228	1.02	(1.01, 1.03)	1.00	(0.97, 1.03)	0.96
Mother’s BMI	297	1.11	(1.03, 1.21)	1224	1.09	(1.05, 1.12)	1.02	(0.94, 1.12)	0.61
Father’s age in years	290	0.95	(0.89, 1.03)	1213	1.00	(0.97, 1.03)	0.95	(0.88, 1.03)	0.23
Father’s weight (kg)	276	1.03	(0.99, 1.06)	1149	1.01	(1.00, 1.03)	1.01	(0.98, 1.059)	0.45
Father’s BMI	276	1.18	(1.06, 1.32)	1146	1.11	(1.06, 1.17)	1.06	(0.95, 1.20)	0.30
Duration of pregnancy in weeks	292	1.05	(0.80, 1.40)	1219	1.01	(0.91, 1.12)	1.04	(0.78, 1.40)	0.78
Breastfeeding time in months	274	1.01	(0.97, 1.06)	1144	1.01	(0.99, 1.04)	1.00	(0.95, 1.05)	0.91
Intro of food in months	263	1.02	(0.96, 1.08)	1098	1.02	(0.99, 1.05)	1.00	(0.94, 1.07)	0.97
Alcohol in pregnancy	295	1.03	(0.42, 2.51)	1246	0.69	(0.44, 1.07)	1.52	(0.56, 4.14)	0.41
Nicotine in pregnancy	296	3.21	(1.45, 7.07)	1246	1.54	(1.08, 2.21)	2.06	(0.86, 4.90)	0.10
Mother’s weight before pregnancy	268	1.03	(0.99, 1.08)	1144	1.03	(1.02, 1.05)	1.00	(0.96, 1.05)	0.90
Mother’s weight at time of birth (kg)	222	1.02	(0.98, 1.06)	901	1.03	(1.02, 1.04)	0.99	(0.95, 1.03)	0.57
Mother’s num of pregnancies	226	1.21	(0.83, 1.75)	892	1.05	(0.90, 1.21)	1.15	(0.77, 1.71)	0.50
Mother’s num of births	183	1.25	(0.66, 2.34)	688	0.98	(0.78, 1.24)	1.27	(0.65, 2.51)	0.49
Mother has elevated cholesterol	186	2.43	(0.46, 12.07)	674	0.70	(0.25, 2.02)	3.32	(0.47, 23.33)	0.23
Mother has elevated bp	196	0.44	(0.06, 3.44)	706	2.00	(0.96, 4.14)	0.21	(0.02, 1.89)	0.16
Mother is overweight	195	2.78	(1.01, 7.38)	708	2.04	(1.19, 3.51)	1.32	(0.42, 4.11)	0.63
Father has elevated cholesterol	166	2.64	(0.65, 10.70)	608	1.01	(0.41, 2.47)	2.47	(0.47, 13.02)	0.29
Father has diabetes	183	4.87	(0.41, 55.66)	687	2.66	(0.85, 8.30)	1.96	(0.13, 29.59)	0.63
Father is overweight	184	2.91	(1.03, 7.89)	685	1.59	(0.90, 2.79)	1.73	(0.54, 5.55)	0.36
Grandparents have elevated bp	149	0.63	(0.21, 1.89)	530	0.72	(0.41, 1.25)	0.86	(0.25, 2.99)	0.82
Grandparents have diabetes	181	1.26	(0.39, 3.45)	636	1.45	(0.85, 2.48)	0.81	(0.24, 2.7)	0.73
Grandparents had a heart attack	179	0.82	(0.26, 2.62)	655	0.6	(0.30, 1.17)	1.42	(0.37, 5.42)	0.61
Grandparents had a stroke	178	2.12	(0.78, 5.75)	649	0.29	(0.10, 0.80)	7.16	(1.70, 30.16)	0.01
Grandparents are overweight	169	1.79	(0.65, 4.24)	629	1.05	(0.64, 1.73)	1.58	(0.55, 4.58)	0.40
Mother’s num of cigarettes day	132	1.03	(0.95, 1.13)	486	1.04	(1.01, 1.08)	0.99	(0.91, 1.09)	0.88
Father’s num of cigarettes day	119	1.14	(1.01, 1.13)	455	1.00	(0.97, 1.03)	1.07	(1.00, 1.15)	0.05
Portions of fruit per day	59	0.38	(0.08, 1.89)	145	0.47	(0.24, 0.91)	0.82	(0.15, 4.63)	0.82
Portions of vegetables per day	59	0.55	(0.09, 3.45)	145	0.58	(0.28, 1.23)	1.03	(0.14, 7.42)	0.98
Number of meals consumed together per day 1	211	0.87	(0.50, 1.50)	759	1.12	(0.82, 1.54)	0.79	(0.41, 1.49)	0.46
School should do more for health	178	0.58	(0.18, 1.91)	679	0.75	(0.36, 1.59)	0.74	(0.18, 3.02)	0.67

**Table A2:** Adjusted available case analysis—full results, obesity.

Baseline Characteristic	Treatment						Treatment Baseline Interaction		
	Intervention			Non-Intervention					
Obesity	*N*	OR	95% CI	*N*	OR	95% CI	ROR	95% CI	*p*
Anthropometric characteristics									
Weight (kg)	290	1.60	(1.34, 1.90)	1168	1.71	(1.54, 1.90)	0.95	(0.77, 1.16)	0.59
Height (cm)	290	1.10	(1.00, 1.20)	1168	1.14	(1.09, 1.20)	0.97	(0.87, 1.07)	0.54
Tricep skin fold (mm)	290	1.35	(1.18, 1.54)	1168	1.75	(1.60, 1.95)	0.76	(0.65, 0.90)	<0.01
Bicep skin fold (mm)	290	1.41	(1.20, 1.65)	1168	1.66	(1.50, 1.83)	0.84	(0.70, 1.01)	0.07
Abdominal (mm)	290	1.42	(1.25, 1.61)	1168	1.51	(1.39, 1.64)	0.93	(0.80, 1.07)	0.30
SIF (mm)	290	1.43	(1.26, 1.63)	1168	1.50	(1.39, 1.62)	0.95	(0.82, 1.10)	0.48
SSF (mm)	290	1.80	(1.79, 1.82)	1168	1.68	(1.67, 1.69)	1.06	(0.85, 1.32)	0.59
Child sum of 4 skin folds	290	1.14	(1.08, 1.19)	1168	1.18	(1.14, 1.22)	0.96	(0.91, 1.01)	0.15
Wasit (cm)	289	1.45	(1.27, 1.66)	1168	1.48	(1.36, 1.60)	0.98	(0.84, 1.13)	0.75
Hip circumference (cm)	289	1.40	(1.21, 1.51)	1168	1.43	(1.34, 1.54)	0.95	(0.83, 1.07)	0.41
Fat-free mass KOPS formula	285	1.82	(1.41, 2.35)	1138	1.81	(1.58, 2.06)	1.07	(0.80, 1.43)	0.66
Fat mass KOPS formula	285	2.10	(1.61, 2.75)	1138	2.56	(2.12, 3.09)	0.82	(0.60, 1.13)	0.22
Child’s fat mass (%)	285	1.30	(1.18, 1.43)	1138	1.33	(1.26, 1.41)	0.97	(0.87 1.09)	0.65
Child’s fat-free mass (%)	285	0.77	(0.70, 0.85)	1138	0.75	(0.71, 0.80)	1.27	(0.92, 1.15)	0.65
Child’s BMI	290	3.52	(2.27, 5.45)	1168	3.83	(2.93, 5.02)	0.91	(0.91, 0.92)	<0.01
Birth weight (kg)	285	2.35	(0.85, 6.50)	1140	1.62	(1.00, 2.63)	1.47	(0.47, 4.57)	0.50
Birth height (cm)	283	1.06	(0.88, 1.28)	1131	1.04	(0.95, 1.14)	1.02	(0.83, 1.26)	0.83
Child’s head circumference at birth	255	1.16	(0.83, 1.62)	1045	1.07	(0.91, 1.26)	1.09	(0.75, 1.58)	0.66
Weight at 1 year (kg)	265	1.42	(0.97, 2.09)	1007	1.74	(1.38, 2.18)	0.84	(0.54, 1.30)	0.43
Height at 1 year (cm)	265	1.01	(0.86, 1.19)	1004	1.09	(1.00, 1.19)	0.93	(0.78, 1.12)	0.47
Weight at 2 years (kg)	254	1.45	(1.12, 1.86)	981	1.68	(1.43, 1.97)	0.87	(0.65, 1.18)	0.37
Height at 2 years (cm)	250	1.04	(0.91, 1.19)	980	1.07	(1.00, 1.14)	0.97	(0.84, 1.13)	0.73
Physical activity characteristics									
TV or PC per day (hours)	126	4.01	(1.44, 11.16)	452	1.37	(0.78, 2.40)	3.04	(0.98, 9.44)	0.05
Outdoor games are a top 3 activity	127	1.52	(0.20, 11.68)	460	0.46	(0.16, 1.32)	3.21	(0.32, 32.15)	0.32
Indoor games are a top 3 activity	127	0.77	(0.07, 7.99)	459	1.25	(0.45, 3.51)	0.61	(0.05, 7.83)	0.71
Weekly sports club activity (hours)	126	0.20	(0.03, 1.40)	455	1.07	(0.74, 1.54)	0.19	(0.03, 1.40)	0.10
Socio-economic characteristics									
SES middle: low	191	1.16	(0.10, 14.08)	702	0.46	(0.17, 1.30)	2.76	(0.19, 40.69)	0.46
SES high: low	191	0.85	(0.08, 9.20)	702	0.35	(0.14, 0.92)	2.50	(0.19, 31.86)	0.48
Father’s Graduation from School—Grade 9:Grade 10	168	1.33	(0.08, 2.25)	642	0.23	(0.05, 1.05)	5.80	(0.23, 144.89)	0.28
Father’s Graduation from School—Grade 9:Grade 12	168	1.64	(0.16, 1.68)	642	0.39	(0.15, 1.00)	4.02	(0.32, 50.24)	0.28
Father is employed	178	0.51	(0.05, 5.36)	662	0.56	(0.16, 2.02)	1.02	(0.07, 14.66)	0.99
Single parent	193	1.96	(0.35, 10.90)	707	1.62	(0.63, 4.16)	1.06	(0.15, 7.60)	0.96
Family’s health characteristics									
Mother’s age (years)	286	0.96	(0.86, 1.08)	1154	0.95	(0.90, 1.00)	1.02	(0.90, 1.15)	0.81
Mother’s height (cm)	287	0.99	(0.91, 1.07)	1149	0.98	(0.94, 1.02)	1.01	(0.92, 1.10)	0.86
Mother’s weight (kg)	286	1.05	(1.01, 1.08)	1136	1.05	(1.03, 1.06)	1.00	(0.96, 1.032)	0.87
Mother’s BMI	286	1.15	(1.05, 1.27)	1132	1.16	(1.11, 1.21)	0.99	(0.90, 1.10)	0.91
Father’s age in years	278	1.03	(0.95, 1.11)	1123	0.97	(0.92, 1.01)	1.07	(0.97, 1.17)	0.18
Father’s height (cm)	276	0.01	(0.00, 13.37)	1097	0.03	(0.001, 1.03)	0.99	(0.92, 1.07)	0.89
Father’s weight (kg)	265	1.00	(0.96, 1.05)	1065	1.05	(1.03, 1.07)	0.96	(0.91, 1.01)	0.10
Father’s BMI	265	1.07	(0.92, 1.24)	1062	1.23	(1.15, 1.32)	0.87	(0.74, 1.02)	0.08
Duration of pregnancy (weeks)	282	1.29	(0.84, 1.92)	1125	1.07	(0.91, 1.27)	1.20	(0.76, 1.90)	0.43
Breastfeeding time months	259	0.97	(0.86, 1.08)	1063	1.03	(1.01, 1.05)	0.94	(0.84, 1.05)	0.29
Intro of food in months	250	0.96	(0.86, 1.07)	1018	1.03	(1.00, 1.07)	0.93	(0.82, 1.06)	0.29
Alcohol in pregnancy	283	0.45	(0.10, 2.05)	1152	0.44	(0.20, 0.98)	1.05	(0.19, 5.76)	0.96
Nicotine in pregnancy	284	2.06	(0.72, 5.87)	1152	1.24	(0.71, 2.18)	1.65	(0.51, 5.37)	0.41
Mother’s weight before pregnancy	260	1.08	(1.03, 1.13)	1051	1.06	(1.04, 1.08)	1.02	(0.97, 1.07)	0.44
Mother’s weight at time of birth (kg)	215	1.07	(1.03, 1.12)	825	1.04	(1.02, 1.06)	1.03	(0.99, 1.08)	0.17
Mother’s num of pregnancies	217	1.08	(0.61, 1.91)	828	1.00	(0.79, 1.25)	1.08	(0.59, 2.00)	0.80
Mother’s num of births	173	1.28	(0.39, 4.15)	640	1.17	(0.86, 1.58)	1.09	(0.32, 3.70)	0.89
Mother has elevated cholesterol	173	10.35	(1.45, 74.10)	628	1.25	(0.27, 5.75)	8.50	(0.70, 103.44)	0.09
Father has elevated cholesterol	152	3.12	(0.29, 33.19)	570	2.33	(0.72, 7.53)	2.00	(0.14 29.19)	0.61
Father has elevated bp	163	2.40	(0.25, 23.25)	611	0.75	(0.17, 3.39)	3.01	(0.19, 47.39)	0.43
Grandparents have diabetes	167	4.84	(0.76, 30.83)	590	2.10	(0.86, 5.14)	2.35	(0.30, 18.61)	0.42
Grandparents had a heart attack	164	5.22	(0.82, 33.28)	610	0.67	(0.22, 2.02)	8.27	(0.92, 74.01)	0.06
Grandparents had a stroke	163	2.65	(0.42, 16.86)	603	1.36	(0.48, 3.84)	2.03	(0.24, 17.28)	0.52
Grandparents are overweight	154	6.62	(0.72, 61.25)	578	1.83	(0.74, 4.55)	3.53	(0.32, 39.50)	0.31
Mother’s num of cigarettes day	126	1.11	(1.00, 1.24)	458	1.02	(0.97, 1.08)	1.11	(0.98, 1.25)	0.11
Father’s num of cigarettes day	115	1.03	(0.94, 1.17)	425	1.02	(0.97, 1.06)	1.03	(0.93, 1.15)	0.58
Portions of vegetables per day	57	1.16	(0.07, 18.65)	133	0.57	(0.16, 2.08)	2.80	(0.12, 64.11)	0.52
School should do more for health	165	0.82	(0.09, 7.35)	633	0.45	(0.16, 1.26)	1.84	(0.16, 21.02)	0.62

## Appendix B

**Table A3:** Variables not included in adjusted available case analysis due to lack of data: overweight and obesity.

Overweight	Obesity
Child’s physical activity estimate	Making music is a top 3 activity
TV or PC per day (hours)	Playing with toys is a top 3 activity
Weekly frequency of training in sports club—three times per week	Child’s physical activity estimate
Mother’s frequency of sports per week	Physical activity of parent estimate
Nutritional education in school	Mother has diabetes
Not able to feed family health food	Father has diabetes
Mother’s alcohol use—daily	Grandparents have elevated bp
Mother’s smoking—twice a week	Mother has professional training
Mother’s smoking—three times a week	Mother graduated from school
Mother has diabetes	Nutritional education in school
Father has elevated blood pressure	Not able to feed family health food
Mother’s frequency of sports per week	TV or PC per day (hours)
Father frequency of sports per week	Weekly frequency of training in a sports club
Parents’ activity is higher on weekends	Mother’s alcohol use
Mother’s most frequent transport	Mother’s smoking
Father’s most frequent transport	Father’s alcohol use
Cooking together, times per week	Mother’s frequency of sports per week
Elbow width (cm)	Father frequency of sports per week
Waist/hip	Mother’s most frequent transport
	Father’s most frequent transport
	Cooking together, times per week
	Elbow width (cm)
	Father had a heart attack

## Appendix C

**Table A4:** Unadjusted available case analysis—full results, overweight.

Baseline Characteristic	Treatment						Treatment Baseline Interaction		
	Intervention			Non-Intervention					
Overweight	*N*	OR	95% CI	*N*	OR	95% CI	ROR	95% CI	*p*
Anthropometric characteristics									
Weight (kg)	302	1.41	(1.25, 1.60)	1263	1.37	(1.30, 1.45)	1.03	(0.91, 1.19)	0.66
Height (cm)	302	1.09	(1.0, 1.17)	1263	1.05	(1.02, 1.09)	1.03	(0.96, 1.12)	0.40
Tricep skin fold (mm)	302	1.26	(1.13, 1.39)	1263	1.33	(1.27, 1.41)	0.94	(0.84, 1.06)	0.31
Bicep skin fold (mm)	302	1.31	(1.15, 1.50)	1263	1.33	(1.25, 1.41)	0.99	(0.86, 1.15)	0.91
Abdominal (mm)	302	1.26	(1.15, 1.38)	1263	1.33	(1.27, 1.39)	0.95	(0.86, 1.05)	0.30
SIF (mm)	302	1.19	(1.09, 1.30)	1263	1.30	(1.24, 1.36)	0.92	(0.83, 1.01)	0.08
SSF (mm)	302	1.45	(1.24, 1.69)	1263	1.41	(1.33, 1.51)	1.02	(0.87, 1.11)	0.79
Child sum of 4 skin folds	302	1.09	(1.05, 1.12)	1263	1.1	(1.09, 1.12)	0.98	(0.95, 1.02)	0.39
Arm circumference (cm)	302	2.03	(1.56, 2.64)	1262	2.14	(1.90, 2.41)	0.95	(0.72, 1.28)	0.73
Wasit (cm)	301	1.27	(1.16, 1.39)	1263	1.28	(1.23, 1.34)	0.99	(0.90, 1.10)	0.89
Hip circumference (cm)	301	1.17	(1.08, 1.27)	1263	1.24	(1.19, 1.29)	0.94	(0.86, 1.03)	0.18
Birth height (cm)	295	1.02	(0.89, 1.16)	1223	0.95	(0.89, 1.00)	1.08	(0.94, 1.25)	0.32
Birth weight (kg)	297	0.86	(0.41, 1.79)	1235	0.88	(0.65, 1.20)	0.98	(0.44, 2.19)	0.95
Child’s head circumference at birth	265	1.04	(0.80, 1.36)	1128	1.00	(0.90, 1.12)	1.04	(0.79, 1.39)	0.78
Weight at 1 year (kg)	275	1.07	(0.74, 1.56)	1088	1.15	(0.97, 1.35)	0.94	(0.62, 1.40)	0.76
Height at 1 year (cm)	275	1.00	(0.87, 1.14)	1085	0.95	(0.89, 1.01)	1.05	(0.91, 1.22)	0.50
Weight at 2 years (kg)	265	1.29	(0.99, 1.69)	1066	1.27	(1.14, 1.42)	1.02	(0.76, 1.36)	0.91
Height at 2 years (cm)	261	1.00	(0.90, 1.12)	1064	0.98	(0.93, 1.03)	1.02	(0.90, 1.15)	0.74
Fat-free mass KOPS formula	297	1.44	(1.18, 1.76)	1233	1.36	(1.25, 1.47)	1.06	(0.86, 1.33)	0.59
Fat mass KOPS formula	297	1.70	(1.41, 2.05)	1233	1.66	(1.52, 1.83)	1.02	(0.83, 1.27)	0.85
Child’s fat-free mass (%)	297	0.85	(0.79, 0.91)	1233	0.88	(0.86, 0.91)	0.96	(0.89, 1.03)	0.30
Child’s fat mass (%)	297	1.18	(1.10, 1.26)	1233	1.13	(1.10, 1.17)	1.04	(0.97, 1.12)	0.30
Child’s BMI	302	2.54	(1.86, 3.47)	1263	2.55	(2.21, 2.95)	1.00	(0.72, 1.43)	0.98
Physical activity characteristics									
Child is a sports club member	209	0.27	(0.11, 0.67)	758	0.67	(0.42, 1.09)	0.40	(0.14, 1.12)	0.08
Weekly sports club activity (hours)	133	0.38	(0.17, 0.85)	484	0.91	(0.70, 1.17)	0.42	(0.16, 0.92)	0.04
TV or PC per day (hours)	132	1.79	(0.75, 4.23)	480	2.00	(1.46, 2.75)	0.89	(0.33, 2.19)	0.81
Cycling is a top 3 activity	133	0.86	(0.21, 3.49)	487	1.32	(0.73, 2.40)	0.65	(0.12, 2.81)	0.58
Romping is a top 3 activity	133	1.73	(0.48, 6.31)	489	1.03	(0.56, 1.88)	1.69	(0.40, 7.24)	0.47
Painting is a top 3 activity	133	0.43	(0.12, 1.58)	489	0.67	(0.36, 1.24)	0.64	(0.15, 2.75)	0.54
Playing with toys is a top 3 activity	133	1.79	(0.48, 6.68)	489	0.95	(0.52, 1.74)	1.88	(0.45, 8.63)	0.39
Outdoor games are a top 3 activity	133	1.56	(0.43, 5.68)	489	1.11	(0.61, 2.01)	1.41	(0.33, 6.02)	0.64
Indoor games are a top 3 activity	133	0.92	(0.23, 3.76)	488	1.09	(0.56, 2.09)	0.85	(0.16, 3.80)	0.84
Swimming is a top 3 activity	133	1.97	(0.47, 8.21)	488	1.06	(0.47, 2.35)	1.86	(0.32, 9.25)	0.46
TV or PC is a top 3 activity (no)	132	0.86	(0.10, 7.28)	489	3.22	(1.73, 6.01)	0.27	(0.01, 1.78)	0.24
Mother’s weekly sports (hours)	131	0.59	(0.31, 1.15)	473	0.84	(0.66, 1.07)	0.71	(0.31, 1.31)	0.34
Father’s weekly sports (hours)	118	0.89	(0.62, 1.27)	435	1.04	(0.92, 1.19)	0.85	(0.53, 1.18)	0.40
Parents physical activity moderate: inactive	130	0.65	(0.07, 5.98)	487	0.52	(0.23, 1.18)	1.24	(0.15, 26.72)	0.86
Parents physical activity active: inactive	130	0.62	(0.05, 7.71)	487	0.51	(0.19, 1.33)	1.21	(0.09, 31.44)	0.89
Sports club—once per week: not a member	75	0.25	(0.05, 1.34)	271	0.42	(0.16, 1.12)	0.60	(0.07, 3.79)	0.60
Sports club—twice per week: not a member	75	0.73	(0.16, 3.30)	271	0.66	(0.23, 1.88)	1.09	(0.16, 6.74)	0.92
Socio-economic characteristics									
SES middle: low	206	0.45	(0.16, 1.31)	748	1.09	(0.58, 2.05)	0.42	(0.12, 1.43)	0.17
SES high: low	206	0.11	(0.03, 0.37)	748	0.54	(0.28, 1.04)	0.20	(0.04, 0.77)	0.02
Mother graduated from school, Grade 10:Grade 9	206	0.16	(0.05, 0.52)	741	0.84	(0.47, 1.48)	0.19	(0.05, 0.67)	0.01
Mother graduated from school, Grade 12:Grade 9	206	0.13	(0.04, 0.43)	741	0.44	(0.23, 0.84)	0.30	(0.07, 1.10)	0.08
Father graduated from school, Grade 10:Grade 9	183	0.98	(0.35, 2.76)	685	1.11	(0.60, 2.03)	0.89	(0.26, 2.95)	0.85
Father graduated from school, Grade 12:Grade 9	183	0.10	(0.02, 0.48)	685	0.45	(0.24, 0.84)	0.23	(0.03, 1.06)	0.08
Mother has professional training	132	0.23	(0.02, 2.41)	486	0.40	(0.20, 0.82)	0.57	(0.06, 12.86)	0.65
Father has professional training	122	0.45	(0.05, 4.20)	461	0.45	(0.17, 1.15)	1.01	(0.11, 22.46)	>0.99
Mother is employed	208	0.81	(0.33, 1.97)	759	0.81	(0.50, 1.30)	1.00	(0.35, 2.74)	>0.99
Father is employed	192	0.29	(0.08, 1.02)	712	0.68	(0.31, 1.49)	0.43	(0.10, 2.03)	0.26
Single parent	208	0.50	(0.11, 2.25)	757	0.99	(0.52, 1.91)	0.50	(0.07, 2.21)	0.41
Family’s health characteristics									
Mother’s age in years	298	0.95	(0.87, 1.04)	1250	0.98	(0.94, 1.01)	0.97	(0.88, 1.07)	0.56
Mother’s height (cm)	299	0.92	(0.87, 0.98)	1244	0.96	(0.94, 0.98)	0.96	(0.90, 1.03)	0.24
Mother’s weight (kg)	297	1.02	(0.99, 1.05)	1228	1.02	(1.01, 1.039)	1.00	(0.97, 1.03)	0.93
Mother’s BMI	297	1.11	(1.03, 1.21)	1224	1.09	(1.05, 1.12)	1.02	(0.94, 1.12)	0.58
Father’s age in years	290	0.95	(0.89, 1.03)	1213	1.00	(0.97, 1.03)	0.95	(0.88, 1.03)	0.23
Father’s height (cm)	288	0.92	(0.87, 0.98)	1185	0.96	(0.94, 0.99)	0.96	(0.90, 1.02)	0.17
Father’s weight (kg)	276	1.03	(0.99, 1.06)	1149	1.01	(1.00, 1.03)	1.01	(0.98, 1.05)	0.46
Father’s BMI	276	1.18	(1.06, 1.31)	1146	1.11	(1.06, 1.17)	1.06	(0.94, 1.20)	0.31
Duration of pregnancy in weeks	292	1.05	(0.80, 1.39)	1219	1.01	(0.91, 1.12)	1.04	(0.79, 1.44)	0.78
Breastfeeding time months	274	1.01	(0.97, 1.06)	1144	1.01	(0.99, 1.04)	1.00	(0.95, 1.04)	0.97
Intro of food in months	263	1.02	(0.96, 1.08)	1098	1.02	(0.99, 1.05)	1.00	(0.93, 1.06)	0.97
Alcohol in pregnancy	295	1.03	(0.42, 2.51)	1246	0.68	(0.44, 1.07)	1.50	(0.52, 3.94)	0.43
Nicotine in pregnancy	296	3.20	(1.45, 7.06)	1246	1.54	(1.08, 2.21)	2.08	(0.86, 4.94)	0.10
Mother’s weight before pregnancy	268	1.03	(0.99, 1.08)	1144	1.03	(1.02, 1.05)	1.00	(0.96, 1.05)	0.88
Mother’s weight at time of birth (kg)	222	1.02	(0.98, 1.06)	901	1.03	(1.02, 1.04)	0.99	(0.95, 1.03)	0.57
Mother’s num of pregnancies	226	1.20	(0.82, 1.74)	892	1.05	(0.91, 1.21)	1.14	(0.74, 1.68)	0.52
Mother’s num of births	183	1.23	(0.65, 2.31)	688	0.99	(0.79, 1.25)	1.24	(0.61, 2.37)	0.53
Mother has elevated cholesterol	186	2.34	(0.46, 11.91)	674	0.70	(0.25, 2.02)	3.32	(0.39, 22.08)	0.23
Mother has elevated bp	196	0.43	(0.06, 3.43)	706	2.00	(0.96, 4.14)	0.22	(0.01, 1.39)	0.17
Mother is overweight	195	2.72	(1.01, 7.37)	708	2.04	(1.19, 3.49)	1.34	(0.42, 4.09)	0.61
Father has elevated cholesterol	166	2.57	(0.64, 10.32)	608	1.00	(0.41, 2.44)	2.56	(0.44, 12.96)	0.26
Father has diabetes	183	4.79	(0.44, 55.66)	687	2.65	(0.85, 8.30)	1.81	(0.07, 25.91)	0.67
Father had a heart attack	183	1.77	(0.20, 15.97)	687	2.14	(0.70, 6.55)	0.83	(0.04, 7.98)	0.88
Father is overweight	184	2.85	(1.03, 7.89)	685	1.59	(0.90, 2.79)	1.80	(0.55, 5.71)	0.32
Grandparents have elevated bp	149	0.63	(0.21, 1.89)	530	0.71	(0.41, 1.25)	0.88	(0.25, 3.07)	0.84
Grandparents have diabetes	181	1.17	(0.39, 3.45)	636	1.45	(0.85, 2.48)	0.81	(0.22, 2.59)	0.73
Grandparents had a heart attack	179	0.82	(0.26, 2.62)	655	0.59	(0.30, 1.17)	1.39	(0.33, 5.04)	0.63
Grandparents had a stroke	178	2.12	(0.78, 5.75)	649	0.29	(0.10, 0.80)	7.44	(1.83, 34.09)	0.01
Grandparents are overweight	169	1.66	(0.65, 4.24)	629	1.05	(0.64, 1.73)	1.58	(0.54, 4.62)	0.40
Mother’s num of cigarettes day	132	1.03	(0.95, 1.13)	486	1.04	(1.01, 1.07)	1.00	(0.90, 1.08)	0.92
Father’s num of cigarettes day	119	1.07	(1.01, 1.14)	455	1.00	(0.97, 1.03)	1.07	(1.00, 1.15)	0.04
Portions of fruit per day	59	0.38	(0.08, 1.90)	145	0.47	(0.24, 0.91)	0.81	(0.12, 4.43)	0.81
Portions of vegetables per day	59	0.56	(0.09, 3.45)	145	0.58	(0.28, 1.23)	0.95	(0.10, 6.09)	0.96
Number of meals consumed together per day	211	0.87	(0.50, 1.50)	759	1.12	(0.82, 1.54)	0.77	(0.41, 1.47)	0.43
School should do more for health	178	0.58	(0.18, 1.91)	679	0.75	(0.36, 1.59)	0.77	(0.19, 3.42)	0.71
Mother drinks alcohol rarely: never	208	0.35	(0.12, 1.01)	756	0.42	(0.24, 0.72)	0.83	(0.25, 2.92)	0.77
Mother drinks alcohol three times per week: never	208	0.06	(0.01, 0.58)	756	0.28	(0.12, 0.69)	0.23	(0.01, 1.86)	0.22
Father drinks alcohol rarely: never	193	0.75	(0.15, 3.76)	709	0.68	(0.33, 1.39)	1.11	(0.21, 8.48)	0.91
Father drinks alcohol three times per week: never	193	0.21	(0.03, 1.69)	709	0.46	(0.2, 1.08)	0.46	(0.04, 4.86)	0.50
Mother’s frequency of sports									
Mother’s most frequent transport									
Number of times they cook together per week									
Waist/hip									
Elbow width (cm)									
Making music is a top 3 activity									
Mother has diabetes									
Father has elevated blood pressure									
Parents’ activity is higher on weekends									
Nutritional education in school									
Child is vegetarian									
Child’s physical activity estimate									
Not able to feed family healthy food									
TV or PC per day									
Weekly training in a sports club									
Mother drinks alcohol daily: never									
Father drinks alcohol daily: never									
Father smokes									

**Table A5:** Unadjusted available case analysis—full results, obesity.

Baseline Characteristic	Treatment						Treatment Baseline Interaction		
	Intervention			Non-Intervention					
Obesity	*N*	OR	95% CI	*N*	OR	95% CI	ROR	95% CI	*p*
Anthropometric characteristics									
Weight (kg)	290	1.56	(1.32, 1.84)	1168	1.67	(1.52, 1.83)	0.93	(0.78, 1.15)	0.48
Height (cm)	290	1.09	(1.00, 1.20)	1168	1.14	(1.09, 1.20)	0.96	(0.87, 1.06)	0.42
Tricep skin fold (mm)	290	1.34	(1.18, 1.52)	1168	1.70	(1.54, 1.87)	0.79	(0.68, 0.93)	<0.01
Bicep skin fold (mm)	290	1.38	(1.19, 1.62)	1168	1.59	(1.46, 1.74)	0.87	(0.73, 1.05)	0.12
Abdominal (mm)	290	1.41	(1.25, 1.59)	1168	1.48	(1.38, 1.59)	0.96	(0.84, 1.11)	0.52
SIF (mm)	290	1.42	(1.26, 1.60)	1168	1.45	(1.36, 1.55)	0.98	(0.86, 1.13)	0.74
SSF (mm)	290	1.77	(1.46, 2.15)	1168	1.65	(1.5, 1.82)	1.07	(0.87, 1.35)	0.53
Child sum of 4 skin folds	290	1.13	(1.08, 1.18)	1168	1.16	(1.13, 1.19)	0.97	(0.93, 1.03)	0.32
Arm circumference (cm)	290	2.13	(1.58, 2.87)	1167	3.38	(2.72, 4.21)	0.63	(0.44, 0.92)	0.01
Wasit (cm)	289	1.45	(1.27, 1.65)	1168	1.47	(1.37, 1.58)	0.99	(0.86, 1.16)	0.85
Hip circumference (cm)	289	1.35	(1.21, 1.50)	1168	1.42	(1.33, 1.51)	0.95	(0.84, 1.09)	0.42
Fat-free mass KOPS formula	285	1.83	(1.42, 2.35)	1138	1.77	(1.56, 2.01)	1.03	(0.79, 1.39)	0.81
Fat mass KOPS formula	285	2.02	(1.57, 2.59)	1138	2.45	(2.07, 2.90)	0.82	(0.62, 1.14)	0.21
Child’s fat-free mass (%)	285	0.77	(0.70, 0.85)	1138	0.76	(0.72, 0.80)	1.02	(0.91, 1.14)	0.68
Child’s fat mass (%)	285	1.29	(1.18, 1.42)	1138	1.32	(1.25, 1.40)	0.98	(0.88, 1.10)	0.68
Child’s BMI	290	3.31	(2.19, 5.01)	1168	3.60	(2.83, 4.57)	0.92	(0.59, 1.56)	0.73
Birth height (cm)	283	1.06	(0.88, 1.27)	1131	1.04	(0.94, 1.14)	1.02	(0.84, 1.26)	0.83
Birth weight (kg)	285	2.35	(0.85, 6.49)	1140	1.61	(0.99, 2.61)	1.46	(0.48, 4.58)	0.51
Child’s head circumference at birth	255	1.17	(0.83, 1.64)	1045	1.07	(0.91, 1.26)	1.09	(0.75, 1.60)	0.65
Weight at 1 year (kg)	265	1.46	(0.99, 2.16)	1007	1.74	(1.39, 2.18)	0.84	(0.53, 1.31)	0.45
Height at 1 year (cm)	265	1.02	(0.86, 1.20)	1004	1.09	(1.00, 1.19)	0.93	(0.78, 1.12)	0.46
Weight at 2 years (kg)	254	1.47	(1.15, 1.87)	981	1.68	(1.44, 1.97)	0.87	(0.65, 1.18)	0.35
Height at 2 years (cm)	250	1.04	(0.91, 1.20)	980	1.07	(1.00, 1.14)	0.97	(0.84, 1.13)	0.74
Physical activity characteristics									
Child is a sports club member	193	0.33	(0.06, 1.67)	709	0.56	(0.26, 1.23)	0.58	(0.09, 3.80)	0.56
Weekly sports club activity (hours)	126	0.20	(0.03, 1.37)	455	1.06	(0.74, 1.53)	0.19	(0.01, 0.89)	0.09
TV or PC per day (hours)	126	3.88	(1.49, 10.07)	452	1.42	(0.84, 2.41)	2.73	(0.99, 9.42)	0.07
Cycling is a top 3 activity	127	0.67	(0.07, 6.61)	458	1.47	(0.59, 3.69)	0.45	(0.02, 4.46)	0.53
Romping is a top 3 activity	127	0.58	(0.06, 5.72)	460	1.04	(0.41, 2.65)	0.55	(0.02, 5.48)	0.64
Painting is a top 3 activity	127	0.43	(0.06, 3.17)	460	0.96	(0.36, 2.57)	0.45	(0.04, 4.66)	0.48
Outdoor games are a top 3 activity	127	1.56	(0.21, 11.47)	460	0.47	(0.17, 1.32)	3.34	(0.32, 35.95)	0.29
Indoor games are a top 3 activity	127	0.72	(0.07, 7.12)	459	1.22	(0.45, 3.27)	0.59	(0.03, 6.06)	0.68
Swimming is a top 3 activity	127	1.53	(0.15, 15.41)	459	1.89	(0.66, 5.41)	0.81	(0.03, 8.76)	0.87
TV or PC is a top 3 activity (no)	126	0.04	(0.00, 0.44)	460	0.83	(0.27, 2.57)	0.05	(0.01, 0.57)	0.03
Mother’s weekly sports (hours)	125	0.82	(0.39, 1.73)	450	1.01	(0.77, 1.33)	0.81	(0.30, 1.54)	0.61
Father’s weekly sports (hours)	113	0.83	(0.46, 1.50)	406	1.04	(0.83, 1.29)	0.80	(0.34, 1.34)	0.49
Socio-economic characteristics									
SES middle: low	191	1.02	(0.09, 11.77)	702	0.43	(0.16, 1.16)	2.36	(0.18, 58.85)	0.52
SES high: low	191	0.72	(0.07, 7.23)	702	0.33	(0.13, 0.84)	2.17	(0.22, 49.71)	0.54
Mother graduated from school, Grade 10:Grade 9	191	0.51	(0.1, 1.19)	696	0.50	(0.21, 1.19)	1.03	(0.15, 7.11)	0.97
Father graduated from school, Grade 10:Grade 9	168	1.23	(0.07, 20.36)	642	0.22	(0.05, 0.98)	5.62	(0.17, 198.58)	0.29
Father graduated from school, Grade 12:Grade 9	168	1.52	(0.15, 15.03)	642	0.38	(0.15, 0.97)	3.98	(0.40, 91.19)	0.27
Mother has professional training	126	0.03	(0.00, 0.24)	458	0.19	(0.07, 0.49)	0.13	(0.01, 1.66)	0.11
Father has professional training	117	0.17	(0.02, 1.87)	430	0.18	(0.05, 0.62)	0.92	(0.07, 23.72)	0.95
Mother is employed	192	0.58	(0.10, 3.25)	710	0.97	(0.44, 2.13)	0.60	(0.07, 3.75)	0.59
Father is employed	178	0.41	(0.05, 3.74)	662	0.54	(0.15, 1.88)	0.77	(0.07, 18.11)	0.84
Single parent	193	2.00	(0.37, 10.78)	707	1.59	(0.62, 4.06)	1.26	(0.15, 8.13)	0.82
Family’s health characteristics									
Mother’s age (years)	286	0.95	(0.85, 1.07)	1154	0.94	(0.89, 1.00)	1.01	(0.89, 1.14)	0.88
Mother’s height (cm)	287	0.98	(0.91, 1.06)	1149	0.98	(0.94, 1.01)	1.00	(0.92, 1.10)	0.91
Mother’s weight (kg)	286	1.05	(1.01, 1.08)	1136	1.05	(1.03, 1.06)	1.00	(0.96, 1.03)	0.89
Mother’s BMI	286	1.15	(1.05, 1.27)	1132	1.16	(1.11, 1.21)	0.99	(0.89, 1.10)	0.90
Father age (years)	278	1.02	(0.94, 1.11)	1123	0.96	(0.92, 1.01)	1.06	(0.96, 1.16)	0.22
Father’s height (cm)	276	0.95	(0.89, 1.02)	1097	0.96	(0.93, 1.00)	0.99	(0.92, 1.07)	0.83
Father’s weight (kg)	265	1.00	(0.95, 1.05)	1065	1.04	(1.02, 1.06)	0.96	(0.91, 1.01)	0.11
Father’s BMI	265	1.08	(0.93, 1.24)	1062	1.24	(1.15, 1.32)	0.87	(0.73, 1.02)	0.09
Duration of pregnancy in weeks	282	1.31	(0.85, 2.00)	1125	1.06	(0.90, 1.25)	1.23	(0.81, 2.01)	0.37
Breastfeeding time months	259	0.96	(0.85, 1.08)	1063	1.03	(1.00, 1.05)	0.94	(0.81, 1.02)	0.29
Intro of food in months	250	0.96	(0.85, 1.09)	1018	1.03	(1.00, 1.07)	0.93	(0.80, 1.03)	0.27
Alcohol in pregnancy	283	0.43	(0.10, 1.93)	1152	0.43	(0.19, 0.95)	1.01	(0.14, 4.86)	0.99
Nicotine in pregnancy	284	2.15	(0.76, 6.08)	1152	1.29	(0.74, 2.25)	1.67	(0.49, 5.31)	0.40
Mother’s weight before pregnancy	260	1.08	(1.04, 1.13)	1051	1.06	(1.04, 1.08)	1.02	(0.97, 1.07)	0.42
Mother’s weight at time of birth (kg)	215	1.07	(1.03, 1.12)	825	1.04	(1.02, 1.06)	1.03	(0.99, 1.08)	0.16
Mother’s num of pregnancies	217	1.08	(0.61, 1.91)	828	1.00	(0.79, 1.25)	1.08	(0.55, 1.90)	0.80
Mother’s number of births	173	1.27	(0.39, 4.15)	640	1.17	(0.86, 1.58)	1.09	(0.31, 3.58)	0.88
Mother has elevated cholesterol	173	9.94	(1.58, 62.57)	628	1.21	(0.27, 5.38)	8.19	(0.71, 99.26)	0.08
Mother has elevated bp	182	1.65	(0.18, 14.90)	660	3.15	(1.13, 8.81)	0.52	(0.02, 4.74)	0.60
Mother is overweight	179	1.36	(0.15, 12.65)	663	2.70	(1.13, 6.44)	0.51	(0.02, 4.37)	0.58
Father has elevated cholesterol	152	3.42	(0.35, 33.60)	570	2.39	(0.76, 7.49)	1.43	(0.06, 15.50)	0.78
Father has elevated bp	163	2.22	(0.24, 20.41)	611	0.75	(0.17, 3.32)	2.94	(0.12, 40.55)	0.43
Father is overweight	170	3.26	(0.52, 20.43)	635	2.44	(0.94, 6.32)	1.34	(0.14, 10.64)	0.78
Grandparents have diabetes	167	4.89	(0.79, 30.38)	590	2.08	(0.80, 5.03)	2.36	(0.31, 21.62)	0.41
Grandparents had a heart attack	164	4.95	(0.80, 30.72)	610	0.67	(0.22, 2.02)	7.35	(0.90, 75.19)	0.07
Grandparents had a stroke	163	2.62	(0.42, 16.38)	603	1.40	(0.50, 3.92)	1.87	(0.20, 15.54)	0.56
Grandparents are overweight	154	6.64	(0.72, 60.94)	578	1.76	(0.72, 4.31)	3.78	(0.44, 82.66)	0.28
Mother’s num of cigarettes day	126	1.11	(1.00, 1.23)	458	1.02	(0.97, 1.08)	1.09	(0.97, 1.23)	0.15
Father’s num of cigarettes day	115	1.04	(0.94, 1.14)	425	1.02	(0.98, 1.06)	1.02	(0.90, 1.12)	0.76
Portions of fruit per day	57	0.36	(0.02, 5.89)	133	2.23	(0.93, 5.34)	0.16	(0.01, 2.93)	0.22
Portions of vegetables per day	57	1.15	(0.07, 18.64)	133	0.57	(0.16, 2.08)	2.02	(0.06, 39.40)	0.65
Number of meals consumed together per day 1	196	0.37	(0.13, 1.05)	710	0.94	(0.56, 1.58)	0.40	(0.12, 1.21)	0.12
School should do more for health	165	0.87	(0.10, 7.60)	633	0.45	(0.16, 1.24)	1.92	(0.22, 42.04)	0.59
Father drinks alcohol rarely: never	179	0.20	(0.03, 1.23)	662	0.44	(0.17, 1.19)	0.45	(0.06, 4.26)	0.45
Father drinks alcohol three times per week: never	179	0.11	(0.01, 1.29)	662	0.13	(0.03, 0.66)	0.82	(0.03, 16.50)	0.90
Father drinks alcohol daily: never									
Mother’s frequency of sports									
Father frequency of sports									
Mother’s most frequent transport									
Number of times they cook together per week									
Elbow width (cm)									
Waist/hip									
Playing with toys is a top 3 activity									
Making music is a top 3 activity									
Mother has diabetes									
Father has diabetes									
Father had a heart attack									
Grandparents have elevated bp									
Nutritional education in school									
Child is vegetarian									
Mother graduated from school, Grade12: Grade 9									
Children’s activity estimate									
Physical activity estimate of parent									
Not able to feed family healthy food									
TV or PC per day									
Sports club frequency									
Mother drinks alcohol									
Mother’s smoking									

## Appendix D

**Table A6:** Analysis from pooled imputed datasets, overweight.

Baseline Characteristic	Treatment Baseline Interaction		
	N = 1565		
Overweight	ROR	95% CI	*p*
Child’s weight (kg)	1.04	(0.91, 1.19)	0.59
Child’s height (cm)	1.04	(0.96, 1.12)	0.36
Tricep skin fold (mm)	0.94	(0.83, 1.05)	0.27
Bicep skin fold (mm)	0.99	(0.85, 1.14)	0.86
Abdominal circumference (mm)	0.94	(0.85, 1.04)	0.23
Suprailiac skinfold (mm)	0.91	(0.83, 1.01)	0.07
Subscapular skinfold (mm)	1.02	(0.86, 1.21)	0.81
Arm circumference (cm)	0.95	(0.71, 1.27)	0.72
Waist (cm)	0.99	(0.90, 1.10)	0.89
Hip circumference	0.94	(0.86, 1.03)	0.18
Elbow width (cm)	1.19	(0.05, 25.62)	0.91
Mother’s age (years)	0.97	(0.89, 1.07)	0.58
Mother’s height (m)	0.02	(0.00, 17.58)	0.27
Mother’s weight (kg)	1.00	(0.97, 1.03)	0.93
Mother’s BMI	1.02	(0.94, 1.12)	0.60
Father’s age (years)	0.96	(0.89, 1.03)	0.25
Father’s height (m)	0.01	(0.00, 7.20)	0.18
Father’s weight (kg)	1.01	(0.98, 1.05)	0.52
Father’s BMI	1.07	(0.95, 1.20)	0.28
Duration of pregnancy (weeks)	1.05	(0.78, 1.42)	0.74
Birth weight (grams)	1.00	(1.00, 1.00)	0.99
Birth height (cm)	1.08	(0.93, 1.25)	0.31
Head circumference at birth	1.02	(0.78, 1.35)	0.87
Weight at one year (grams)	1.00	(1.00, 1.00)	0.48
Height at one year (cm)	1.02	(0.88, 1.17)	0.80
Weight at two years (grams)	1.00	(1.00, 1.00)	0.66
Height at two years (cm)	0.99	(0.89, 1.12)	0.92
Breastfeeding time (months)	1.00	(0.95, 1.05)	1.00
Introduction to food (months)	1.01	(0.95, 1.07)	0.79
Alcohol use during pregnancy	1.55	(0.57, 4.21)	0.39
Nicotine use during pregnancy	2.12	(0.89, 5.06)	0.09
Mother’s weight before pregnancy	0.99	(0.95, 1.03)	0.72
Mother’s weight at the time of birth (kg)	0.99	(0.95, 1.02)	0.41
Weight change during pregnancy	0.97	(0.91, 1.03)	0.38
Weight change during breastfeeding	1.02	(0.93, 1.12)	0.65
Number of pregnancies	1.02	(0.65, 1.61)	0.91
Number of births	1.05	(0.53, 2.07)	0.90
Number of miscarriages	0.95	(0.43, 2.09)	0.89
Single parent	0.51	(0.14, 1.89)	0.31
Mother’s graduation from school, Grade 9:Grade 10	0.27	(0.05, 1.33)	0.10
Mother’s graduation from school, Grade 12:Grade 10	0.36	(0.05, 2.42)	0.28
Father’s graduation from school, Grade 9:Grade 10	1.01	(0.34, 2.99)	0.98
Father’s graduation from school, Grade 12:Grade 10	0.29	(0.06, 1.32)	0.11
Mother completed professional training	0.71	(0.01, 52.27)	0.86
Father completed professional training	0.75	(0.07, 7.97)	0.80
Mother is employed	0.77	(0.25, 2.37)	0.65
Father is employed	0.57	(0.11, 3.03)	0.49
Number of adults in household	1.43	(0.37, 5.55)	0.59
Number of children in household	1.05	(0.54, 2.04)	0.88
TV + PC per day (hours)	1.08	(0.43, 2.75)	0.86
Cycling is a top 3 activity	1.37	(0.18, 10.59)	0.75
Romping is a top 3 activity	1.00	(0.18, 5.59)	1.00
Painting is a top 3 activity	1.16	(0.12, 11.63)	0.89
Playing with toys is a top 3 activity	0.91	(0.13, 6.48)	0.92
Playing outdoors a top 3 activity	1.03	(0.2, 5.37)	0.98
Playing indoors a top 3 activity	0.76	(0.12, 4.83)	0.76
Swimming is a top 3 activity	1.57	(0.2, 12.52)	0.66
TV or PC is a top 3 activity (no)	0.88	(0.06, 12.47)	0.92
Child is a sports club member	0.53	(0.14, 1.97)	0.33
Weekly sports club activity (hours)	0.57	(0.13, 2.42)	0.41
Sports club frequency—once per week: never	0.91	(0.07, 11.81)	0.94
Sports club frequency—twice per week: never	1.14	(0.07, 1.97)	0.92
Mother has elevated cholesterol	1.00	(0.16, 6.14)	1.00
Mother has elevated blood pressure	1.10	(0.16, 7.52)	0.92
Mother is overweight	1.74	(0.35, 8.73)	0.48
Father has elevated cholesterol	1.37	(0.31, 6.02)	0.67
Father has elevated blood pressure	0.59	(0.07, 4.8)	0.61
Father has diabetes	1.34	(0.15, 11.6)	0.78
Father had a heart attack	0.98	(0.10, 9.61)	0.98
Father is overweight	1.43	(0.43, 4.74)	0.55
Grandparents have elevated cholesterol	1.69	(0.45, 6.28)	0.42
Grandparents have elevated blood pressure	1.05	(0.26, 4.24)	0.94
Grandparents have diabetes	0.93	(0.16, 5.48)	0.93
Grandparents had a heart attack	1.35	(0.24, 7.54)	0.72
Grandparents had a stroke	2.27	(0.45, 11.56)	0.31
Grandparents overweight	1.59	(0.53, 4.79)	0.40
Mother’s alcohol use—rarely: never	0.89	(0.21, 3.7)	0.87
Mother’s alcohol use—three times per week: never	0.33	(0.02, 5.62)	0.43
Mother’s number of cigarettes per day	1.00	(0.93, 1.07)	1.00
Father’s alcohol use—rarely: never	1.78	(0.35, 9.16)	0.49
Father’s alcohol use—daily: never	0.94	(0.10, 0.55)	0.96
Father’s nicotine use	1.89	(0.13, 27.35)	0.63
Father’s number of cigarettes per day	1.03	(0.98, 1.08)	0.29
Parents’ physical activity—moderately active: inactive	0.93	(0.09, 9.37)	0.95
Mother’s sports per week (hours)	0.81	(0.42, 1.55)	0.50
Father’s sports per week (hours)	0.93	(0.63, 1.36)	0.70
Father: frequency of sports per week—twice: never	0.52	(0.06, 4.77)	0.56
Portions of fruit per day	0.77	(0.34, 1.78)	0.54
Portions of vegetables per day	0.84	(0.30, 2.36)	0.72
Vegetarian	0.33	(0.12, 0.88)	0.03
Number of meals eaten together per day	0.98	(0.48, 2.01)	0.97
Sum of the 4 skin folds	0.98	(0.94, 1.02)	0.36
SES—middle: low	0.49	(0.15, 1.63)	0.24
SES—high: low	0.27	(0.05, 1.53)	0.13
Fat-free mass—KOPS formula	1.10	(0.89, 1.37)	0.38
Fat mass—KOPS formula	1.03	(0.83, 1.27)	0.79
Fat-free mass (%)	0.96	(0.89, 1.03)	0.28
Fat mass (%)	1.04	(0.96, 1.12)	0.32
Child’s BMI	0.99	(0.71, 1.40)	0.98
Child’s BMI-SDS	1.05	(0.49, 2.23)	0.91
Number of living rooms	0.92	(0.49, 1.73)	0.79
Agreed school should do more for health education	2.00	(0.58, 6.94)	0.27
Child height (m) Waist/hip Child’s frequency of TV or PC per day			
Playing music as a top3 activity Child’s physical activity estimated by parent			
Frequency of sports club per week—three: never			
Mother has diabetes			
Mother had a heart attack			
Mother had a stroke			
Father had a stroke			
Mother’s alcohol use—daily: never			
Mother’s smoking			
Parents’ physical activity is higher on the weekend			
Mother’s frequency of sports per week			
Father’s frequency of sports per week: three times: never			
Mother’s most frequently used form of transportation			
Father’s most frequently used form of transportation			
Bath is available			
Number of times they cook together per week			
Nutritional education in elementary school			
Pay attention to the health of your family at home			
Concerned about not being able to buy enough food			
Not able to feed the family healthy food due to financial reasons			

**Table A7:** Analysis from pooled imputed datasets, obesity.

Baseline Characteristic	Treatment Baseline Interaction		
	N = 1458		
Obesity	ROR	95% CI	*p*
Child’s weight (kg)	0.95	(0.77, 1.16)	0.59
Child’s weight (cm)	0.97	(0.87, 1.07)	0.54
Tricep skin fold (mm)	0.76	(0.65, 0.90)	<0.01
Bicep skin fold (mm)	0.84	(0.70, 1.01)	0.07
Abdominal circumference (mm)	0.93	(0.80, 1.07)	0.30
Suprailiac skinfold (mm)	0.95	(0.82, 1.10)	0.47
Subscapular skinfold (mm)	1.06	(0.85, 1.32)	0.59
Arm circumference (cm)	0.61	(0.42, 0.89)	0.01
Waist (cm)	0.98	(0.84, 1.13)	0.75
Hip circumference (cm)	0.95	(0.89, 1.00)	0.06
Elbow width (cm)	1.01	(0.06, 15.61)	1.00
Mother’s age (years)	1.02	(0.90, 1.15)	0.77
Mother’s weight (kg)	1.00	(0.96, 1.03)	0.86
Mother’s BMI	0.99	(0.90, 1.10)	0.88
Father’s age (years)	1.07	(0.97, 1.17)	0.17
Father’s weight (kg)	0.96	(0.91, 1.01)	0.13
Father’s BMI	0.88	(0.75, 1.04)	0.13
Duration of pregnancy (weeks)	1.20	(0.76, 1.90)	0.44
Birth weight (grams)	1.00	(1.00, 1.00)	0.48
Birth height (cm)	1.03	(0.83, 1.26)	0.81
Head circumference at birth	1.09	(0.76, 1.58)	0.64
Weight at one year (grams)	1.00	(1.00, 1.00)	0.40
Height at one year (cm)	0.93	(0.78, 1.12)	0.46
Weight at two years (grams)	1.00	(1.00, 1.00)	0.50
Height at two years (cm)	0.98	(0.85, 1.13)	0.78
Breastfeeding time (months)	0.96	(0.88, 1.05)	0.36
Introduction to food (months)	0.95	(0.84, 1.07)	0.37
Alcohol use during pregnancy	1.05	(0.19, 5.79)	0.95
Nicotine use during pregnancy	1.65	(0.51, 5.38)	0.41
Mother’s weight before pregnancy	1.02	(0.98, 1.07)	0.34
Mother’s weight at the time of birth (kg)	1.02	(0.98, 1.07)	0.28
Weight change during pregnancy	1.06	(0.98, 1.16)	0.15
Weight change during breastfeeding	0.99	(0.88, 1.11)	0.83
Number of pregnancies	1.18	(0.68, 2.05)	0.54
Number of births	1.27	(0.58, 2.79)	0.55
Number of miscarriages	1.05	(0.27, 3.99)	0.95
Child takes medication	1.61	(0.22, 11.68)	0.63
Single parent	0.72	(0.13, 3.97)	0.70
Mother’s graduation from school, Grade 9:Grade 10	1.24	(0.22, 7.01)	0.80
Mother’s graduation from school, Grade 12:Grade 10	0.72	(0.10, 5.15)	0.74
Father’s graduation from school, Grade 9:Grade 10	1.17	(0.13, 10.68)	0.89
Father’s graduation from school, Grade 12:Grade 10	1.39	(0.25, 7.66)	0.70
Mother completed professional training	0.57	(0.08, 3.89)	0.56
Father completed professional training	1.26	(0.13, 11.94)	0.84
Mother is employed	1.00	(0.23, 4.26)	1.00
Father is employed	1.21	(0.29, 5.01)	0.79
Number of adults in household	1.38	(0.19, 9.78)	0.74
Number of children in household	1.35	(0.64, 2.81)	0.42
TV + PC per day (hours)	1.29	(0.62, 2.68)	0.49
Cycling is a top 3 activity	0.66	(0.13, 3.41)	0.62
Romping is a top 3 activity	1.25	(0.26, 5.99)	0.77
Painting is a top 3 activity	0.69	(0.13, 3.74)	0.66
Playing outdoors a top 3 activity	1.57	(0.39, 6.38)	0.52
Playing indoors a top 3 activity	0.55	(0.10, 3.17)	0.49
Swimming is a top 3 activity	1.16	(0.21, 6.51)	0.86
TV or PC is a top 3 activity (no)	0.38	(0.04, 3.91)	0.40
Child is a sports club member	0.61	(0.12, 3.07)	0.54
Weekly sports club activity (hours)	0.85	(0.50, 1.44)	0.53
Mother has elevated cholesterol	1.75	(0.36, 8.38)	0.48
Mother has elevated blood pressure	0.92	(0.23, 3.61)	0.90
Mother is overweight	0.98	(0.22, 4.3)	0.98
Father has elevated cholesterol	0.89	(0.22, 3.57)	0.87
Father has elevated blood pressure	1.53	(0.33, 7.06)	0.58
Father is overweight	0.75	(0.16, 3.45)	0.70
Grandparents have elevated cholesterol	1.24	(0.27, 5.74)	0.79
Grandparents have elevated blood pressure	1.75	(0.32, 9.63)	0.51
Grandparents have diabetes	1.43	(0.35, 5.72)	0.61
Grandparents had a heart attack	2.05	(0.58, 7.29)	0.27
Grandparents had a stroke	1.21	(0.30, 4.85)	0.79
Grandparents are overweight	1.33	(0.37, 4.86)	0.66
Mother’s number of cigarettes per day	1.05	(0.98, 1.13)	0.15
Father’s alcohol use—rarely: never	0.88	(0.14, 5.75)	0.90
Father’s alcohol use —three time a week: never	0.95	(0.07, 13.58)	0.97
Father’s number of cigarettes per day	1.01	(0.96, 1.06)	0.79
Parents’ activity is higher on the weekend	1.07	(0.08, 13.97)	0.96
Mother’s weekly sports (hours)	0.93	(0.49, 1.76)	0.80
Father’s weekly sports (hours)	0.92	(0.67, 1.26)	0.60
Portions of fruit per day	0.99	(0.45, 2.16)	0.97
Portions of vegetables per day	1.17	(0.64, 2.13)	0.61
Vegetarian	1.11	(0.27, 4.47)	0.88
Number of meals eaten together per day	0.79	(0.37, 1.70)	0.54
Sum of the 4 skin folds	0.96	(0.91, 1.01)	0.14
SES—middle: low	1.68	(0.19, 14.69)	0.63
SES—middle: low	1.61	(0.24, 10.73)	0.62
Fat-free mass—KOPS formula	1.07	(0.80, 1.42)	0.65
Fat mass—KOPS formula	0.84	(0.62, 1.13)	0.25
Fat-free mass (%)	1.02	(0.91, 1.14)	0.78
Fat mass (%)	0.97	(0.87, 1.09)	0.65
Child’s BMI	0.91	(0.55, 1.49)	0.70
Child’s BMI-SDS	0.65	(0.19, 2.23)	0.50
Number of living rooms	0.96	(0.48, 1.91)	0.90
Nutritional education in elementary school	0.85	(0.27, 2.65)	0.78
Agreed school should do more for health education	0.99	(0.22, 4.57)	0.99
Waist/hip Child’s height (m) Child’s frequency of TV or PC per day Playing with toys is a top 3 activity Playing music is a top 3 activity Child’s physical activity estimated by parent			
Frequency of sports club attendance per week			
Mother has diabetes			
Mother had a heart attack			
Mother had a stroke			
Father has diabetes			
Father had a heart attack			
Father had a stroke			
Mother’s alcohol use			
Mother’s smoking			
Father’s alcohol use—daily: never			
Father’s smoking			
Mother’s frequency of sports per week			
Father frequency of sports per week:			
Mother’s most frequently used form of transportation			
Father’s most frequently used form of transportation			
Alternative diet Net household income Bath is available Number of times they cook together per week Concern about not being able to buy enough food Not able to feed the family healthy food due to financial reasons Physical activity estimate of parent			

**Table A8:** Baseline variables excluded from MICE calculations due to collinearity or no data.

Excluded Baseline Characteristic
Reactance
Resistance
Medication in pregnancy
Complications in pregnancy
Illnesses in pregnancy
Mother’s weight gain during pregnancy
Twins
Mother’s cholesterol level
Father’s cholesterol level
Grandparents’ cholesterol levels
