# Supplementary material for: Exploring the Effect of an Obesity-Prevention Intervention on Various Child Subgroups: A Post Hoc Subgroup Analysis of the Kiel Obesity Prevention Study
Source: Nutrients. 2024 Sep 23;16(18):3220. doi: 10.3390/nu16183220 (PMC11434954; doi:10.3390/nu16183220)
Supplement: Supplementary file 1 [file nutrients-16-03220-s001.zip › nutrients-3207081-supplementary.pdf]

Table S1 Difference in Combined Variable Importance Scores Between Intervention Groups When Predicting Overweight

**Overweight**	**Difference between intervention groups**
Father hours of sport week	3.91
Tricep skin fold (mm)	3.67
Child physical activity estimate	3.35
Father frequency of sports week	3.35
Father Overweight	3.31
Arm circumference (cm)	3.04
SES	2.61
Mother number of pregnancies	2.61
Mother weight before pregnancy (kg)	2.53
Elbow width (cm)	2.44
Father Heart attack	2.24
Portions of fruit per day	2.20
Grandparents Overweight	2.18
TV or PC per day	2.13
Fat used for cooking	2.12
Mother number of miscarriages	2.05
Mother frequency of sports per week	2.05
Waist (cm)	1.96
SSF (mm)	1.92
Making music a top 3 activity	1.69
Child age	1.61
Father most frequent transport	1.61
Mother Diabetes	1.59
Indoor games a top 3 activity	1.56
Romping a top 3 activity	1.55
Grandparents Diabetes	1.47
Mothers' graduation from school	1.43
Mother elevated cholesterol	1.42
Father Stroke	1.42
Intro of food (months)	1.36
Grandparents elevated blood pressure	1.33
Mother most frequent transport	1.31
Not able to feed family healthy food	1.28
Mother age (years)	1.28
Grandparents Heart attack	1.23
Father Alcohol	1.13
Duration of pregnancy (weeks)	1.02
Bath available	1.00
Mother employed	1.00
Father BMI	0.88
Painting a top 3 activity	0.86
Mother heart attack	0.76
Mother height (m)	0.75

Child taking medication	0.57
Child BMI SDS	0.50
Father smoking	0.49
Concern for not able to buy enough food	0.42
Child head circumference at birth	0.38
Child BMI	0.38
Frequent activity playing with toys	0.37
Weekly training in sports club	0.35
Mother Overweight	0.34
Portions of vegetables per day	0.33
Parent activity higher on weekends	0.30
Swimming a top 3 activity	0.30
Mother weight (kg)	0.29
Father employed	0.17
Mother elevated blood pressure	0.13
Number of children in household	0.08
Father height in m	0.06
Father Diabetes	0.04
Fat mass KOPS formula	0.01
Mother number of cigarettes per day	0.01
Mother Stroke	0.00
Mother professional training	-0.02
Nicotine in pregnancy	-0.05
Father Num of cigarettes per day	-0.09
Abdominal skin fold (mm)	-0.10
Alcohol in pregnancy	-0.12
Father professional training	-0.24
Frequent activity cycling	-0.26
Father weight (kg)	-0.33
Grandparents elevated cholesterol	-0.42
Intervention group	-0.48
TV or PC a top 3 activity	-0.48
Mother BMI	-0.62
Physical activity estimate of parent	-0.65
Fat used for bread spread	-0.66
Adults in household	-0.72
Mother num of births	-0.83
SIF (mm)	-0.84
Mother Weight change in breastfeeding	-0.85
Child vegetarian	-0.91
TV or PC per day (hours)	-0.94
Cooking together	-0.98
Father age years	-1.13
Weekly sports club activity (hours)	-1.14
Bicep skin fold (mm)	-1.24
Outdoor games a top 3 activity	-1.33
Sum of 4 skin folds (mm)	-1.34
Grandparents Stroke	-1.46
Child sports club member	-1.47

Father elevated cholesterol	-1.48
Father elevated blood pressure	-1.51
Breastfeeding time (months)	-1.51
Mother Smoking	-1.62
Fathers graduation from school	-1.69
School	-1.72
Mother Weight change in pregnancy	-1.76
Waist/hip	-1.81
Mother Alcohol	-1.92
Weight at 1 year (grams)	-1.96
Single parent	-1.98
Birth weight grams	-1.99
Child alternative diet	-2.00
Mother sport per week (hours)	-2.05
Hip circumference (cm)	-2.48
Should school do more for health education	-2.62
Net household income	-2.62
Height (cm)	-2.68
Sex	-2.74
Fat free mass KOPS formula	-2.77
Mother Weight kg at time birth	-3.14
Child height (m)	-3.34
Birth height (cm)	-3.88
Child fat-free mass percentage	-3.90
Number of living rooms	-4.09
Weight (kg)	-5.28
Height at 1year (cm)	-5.92
Height at 2years (cm)	-7.51
Father sport per week (hours)	3.91
Tricep skin fold (mm)	3.67

Table S2 Difference in Combined Variable Importance Scores Between Intervention Groups When Predicting Obesity

**Obesity**	**Difference between intervention groups**
Arm circumference (cm)	6.08
Portions of vegetables per day	5.18
Waist (cm)	5.15
SSF (mm)	4.83
TV or PC per day	4.81
Fat free mass KOPS formula	4.56
Mother Diabetes	3.99
Abdominal skin fold (mm)	3.74
Mother weight before pregnancy (kg)	3.42
Swimming a top 3 activity	3.42
Father Alcohol	3.37
Child BMI	3.27
Tricep skin fold (mm)	3.11
Father frequency of sports per week	3.05
Fat mass KOPS formula	3.04
Indoor games a top 3 activity	2.97
Weight (kg)	2.90
Father hours of sport per week	2.87
Fathers' graduation from school	2.86
Elbow width (cm)	2.72
Alcohol in pregnancy	2.68
Mother num of miscarriages	2.67
Child sports club member	2.60
Child BMI SDS	2.58
Grandparents Stroke	2.57
Mother Weight change in pregnancy	2.53
Sum of 4 skin folds (mm)	2.45
Portions of fruit per day	2.44
Single parent	2.42
Making music a top 3 activity	2.36
Weight at 2 years (grams)	2.25
Mother BMI	2.17
Father Overweight	1.91
Cycling a top 3 activity	1.83
Not able to feed family healthy food	1.79
Mother Overweight	1.63
Mother elevated blood pressure	1.63
Duration of pregnancy (weeks)	1.55
Father smoking	1.41
Father height (m)	1.36
Mother employed	1.30
Romping a top 3 activity	1.29
Child vegetarian	1.24
Number of children in household	1.20

Mother weight (kg)	1.10
Child Fat-free mass percentage	1.05
Mother Smoking	1.04
Physical activity estimate of parent	1.02
Parent activity higher on weekends	1.00
Mother most frequent transport	0.99
Father BMI	0.93
Grandparents Diabetes	0.90
Mother age (years)	0.89
Father most frequent transport	0.89
Father professional training	0.88
Mother weight change in breastfeeding (kg)	0.67
Mother frequency of sports week	0.61
Child Alternative diet	0.61
Father elevated cholesterol	0.60
Fat used for cooking	0.52
TV or PC a top 3 activity	0.42
Bath available	0.41
Father employed	0.39
Breastfeeding time (months)	0.33
Bicep skin fold (mm)	0.18
Child physical activity estimate	0.17
Height (cm)	0.11
Mother professional training	0.11
Father Stroke	0.00
SIF (mm)	-0.04
Father weight (kg)	-0.16
Mother elevated cholesterol	-0.17
School	-0.19
Outdoor games a top 3 activity	-0.19
Waist/hip	-0.20
Number of living rooms	-0.25
Net household income	-0.29
Mother sports per week (hours)	-0.32
Nicotine in pregnancy	-0.34
Grandparents elevated blood pressure	-0.41
Mother number of cigarettes per day	-0.41
Intro of food (months)	-0.41
Painting a top 3 activity	-0.42
Grandparents overweight	-0.53
Father age (years)	-0.57
TV or PC per day (hours)	-0.59
Mothers' graduation from school	-0.67
Birth height (cm)	-0.71
Birth weight (grams)	-0.73
SES	-0.75
Concern for not able to buy enough food	-0.76
Adults in household	-0.76
Hip circumference (cm)	-0.81

Weekly sports club activity (hours)	-0.82
Weekly frequency of sports club	-0.84
Father Diabetes	-0.84
Playing with toys a top 3 activity	-0.85
Mother number of births	-0.89
Mother number of pregnancies	-1.02
Intervention group	-1.16
Fat used for bread spread	-1.33
Child taking medication	-1.36
Nutritional education in elementary school	-1.44
Child sex	-1.64
Child age	-1.78
Grandparents Heart attack	-1.83
Father Heart attack	-1.86
Mother Stroke	-2.00
Father elevated blood pressure	-2.00
Should school do more for health education	-2.01
Father number of cigarettes per day	-2.40
Mother weight at time of birth (kg)	-2.42
Child head circumference at birth (cm)	-2.45
Grandparents elevated cholesterol	-2.69
Height at 2 years (cm)	-2.70
Child height (m)	-2.71
Cooking together	-2.92
Mother Alcohol	-3.00
Mother Heart attack	-3.37
Mother height in (m)	-3.41
Height at 1 year (cm)	-3.54
Weight at 1 year (grams)	-7.79
